# Engineered human plasma-derived cryogels as a multifunctional scaffold to promote diabetic wound healing

**DOI:** 10.1016/j.mtbio.2026.103151

**Published:** 2026-04-22

**Authors:** Yueming Zhao, Jiajun Hu, Kairui Duan, Tingting Li, Mian Lin, Bae Hoon Lee

**Affiliations:** a School of Pharmaceutical Sciences, Zhejiang Chinese Medical University, Hangzhou, Zhejiang, 311402, China; b Zhejiang Key Laboratory of Soft Matter Biomedical Materials, Wenzhou Institute, University of Chinese Academy of Sciences, Wenzhou, Zhejiang, 325000, China; c Department of Periodontics, School & Hospital of Stomatology, Wenzhou Medical University, Wenzhou, Zhejiang, 325027, China

**Keywords:** Human plasma, Engineered cryogels, Endogenous VEGF, Diabetic wounds, Multifunctional properties

## Abstract

Chronic wounds, characterized by prolonged inflammation and impaired tissue regeneration, represent a critical clinical challenge, particularly in diabetic patients. Conventional therapies often fail to address the multifaceted demands of healing, which requires integrated structural support, bioactive signaling, and immune modulation. To overcome these limitations, we engineered a macroporous cryogel scaffold from methacryloyl-modified human plasma (PlasmaMA) via cryopolymerization. This process transforms the native bioactivity of plasma into a stable, biomimetic extracellular matrix (ECM)-like architecture with tunable mechanical resilience and degradability. The resulting PlasmaMA cryogel inherently retains essential human ECM proteins (e.g., albumin, fibronectin, fibrinogen) and growth factors (e.g., VEGF), which collectively facilitate robust cell adhesion, sustain pro-regenerative signaling, and promote angiogenesis. *In vitro*, the scaffold demonstrates potent antioxidant activity and attenuates pro-inflammatory responses in macrophages. In a diabetic rat wound model, the PlasmaMA cryogel significantly accelerated wound closure, enhanced collagen deposition and maturation, and improved functional neovascularization compared to scaffolds derived from bovine or human serum albumin. By converging native bioactivity with engineered material properties, the PlasmaMA cryogel creates a pro-healing microenvironment that actively orchestrates tissue repair. This work establishes a versatile human plasma-derived platform as a promising multifunctional therapy for complex wound healing.

## Graphical abstract

The human plasma-derived methacrylated cryogel leverages intrinsic VEGF to promote angiogenesis, while diverse plasma components synergistically confer antioxidant, anti-inflammatory, and pro-collagen deposition effects. By integrating immune modulation with tissue repair, this cryogel presents a promising therapeutic strategy for the treatment of chronic diabetic wounds.

**Figure:**
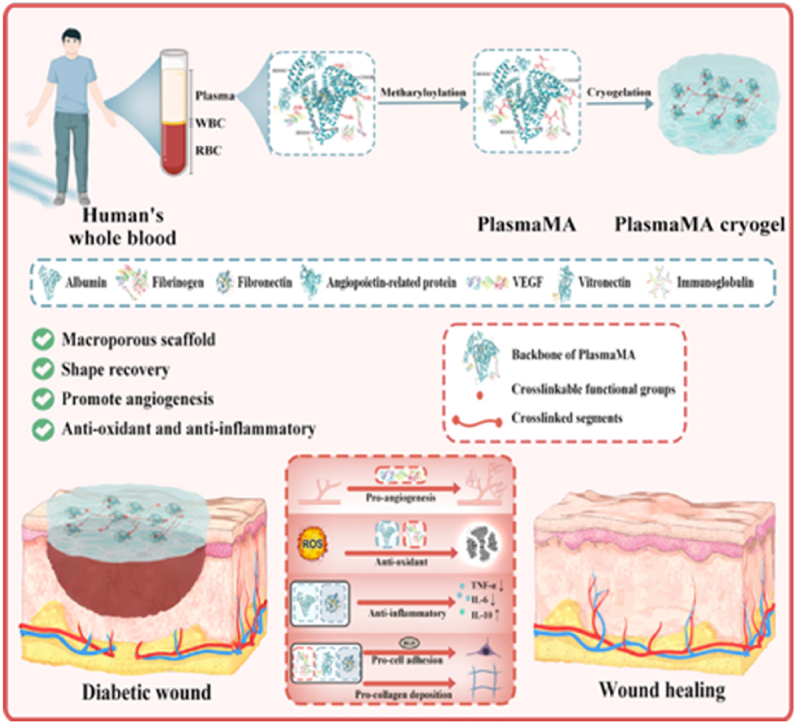

## 1 Introduction

The design of biomaterial scaffolds that support tissue regeneration represents a cornerstone of tissue engineering, particularly in the context of chronic, progressive diseases [1]. Conditions such as diabetes, chronic inflammatory disorders, and ischemic pathologies often result in persistent tissue damage and functional decline, where conventional therapies lack pro-regenerative efficacy [2]. To address these challenges, biomaterial scaffolds, as a fundamental element of tissue engineering, have emerged as a key strategy in promoting effective tissue regeneration. An ideal scaffold is characterized by both biocompatibility and mechanical integrity, along with the capacity to promote cellular adhesion, proliferation, and differentiation for effective tissue reconstruction [3,4].

Protein-based biomaterials offer tunable physical properties and diverse biological functions, with intrinsic biocompatibility and biodegradability that underscore their promise for broad biomedical applications [5,6]. Among these, albumin-based biomaterials (bovine serum albumin (BSA) and human serum albumin (HSA)) exhibit commendable cytocompatibility [7,8] and intrinsic antioxidant capabilities [9,10]. However, they lack pro-angiogenic potential and fail to significantly enhance tissue repair and regeneration. Moreover, BSA, as a xenogeneic protein, carries an inherent risk of eliciting immune rejection, while HSA, although homologous, is constrained by its single-component composition and the absence of synergistic interactions among diverse bioactive molecules. The incorporation of growth factors into biomaterials to stimulate cell proliferation, wound healing, and tissue regeneration has garnered substantial interest[[11], [12], [13]]. Nevertheless, under physiological conditions, a single growth factor proves insufficient to address the multifaceted requirements of tissue repair [14].

Platelet-rich plasma (PRP) has therefore been widely investigated for wound healing because of its enrichment in platelet-derived growth factors. Nevertheless, PRP preparation typically requires additional platelet concentration and activation steps, and its composition and biological activity may vary depending on donor condition, platelet count, and processing procedures [15,16]. Moreover, PRP-based systems are often developed primarily as growth factor-enriched biologic formulations rather than structurally stable scaffold materials.

In contrast, human plasma contains a broad spectrum of native protein components, including albumin, fibrinogen, fibronectin, vitronectin, and other plasma proteins [17,18]. This complex native protein background may provide advantages for cell-material interactions and tissue repair-related biofunctionality, making human plasma a promising precursor for regenerative biomaterials. In addition, plasma is relatively accessible and practical as a starting material for biomaterial preparation[[19], [20], [21], [22], [23], [24], [25], [26]]. However, translating human plasma into high-performance scaffolds remains challenging due to limitations in structural control, cross-linker toxicity, and loss of bioactivity during traditional fabrication methods like glutaraldehyde cross-linking [27,28].

Recent advances in methacryloyl (MA) modification and photocrosslinking offer a promising solution. This strategy, widely applied to natural macromolecules (e.g., gelatin, silk, alginate) [29,30], enables precise spatiotemporal control over scaffold architecture while preserving biological activity. When applied to human plasma, MA modification could yield autologous scaffolds with superior immunocompatibility, minimizing risks of immune rejection and chronic inflammation [31]. Furthermore, plasma-derived materials retain donor-specific biological profiles, aligning with personalized medicine and precision tissue engineering.

ECM, primarily composed of proteins and polysaccharides, is a non-cellular component that plays a pivotal role in regulating cell behavior. It governs key biological processes such as cell adhesion, migration, mechanical support, and modulation of the local microenvironment. Within the ECM, adhesive glycoproteins mediate cell–matrix interactions; proteoglycans confer superior buffering capacity and hydration; and structural proteins impart tensile strength and elastic resilience [32,33]. In addition to its biochemical composition, the ECM possesses several critical biophysical properties—including porosity, appropriate mechanical robustness, high water content, and susceptibility to enzymatic degradation [34]. These ECM properties must be replicated in synthetic scaffolds to guide cellular behavior effectively.

Cryogels represent a class of three-dimensional hydrogels cross-linked at subzero temperatures, offering unique structural and multifunctional properties distinct from traditional hydrogels [35]. Their interconnected porous architecture provides an extensive surface area and ample space, facilitating cell adhesion and migration. This structural configuration closely mimics the natural ECM, furnishing the requisite physical support and growth space for cells [36]. The porous structure not only fosters cell aggregation and proliferation but also enhances nutrient exchange and waste removal [37], thereby accelerating cellular growth and promoting tissue repair. These advantageous properties render cryogels highly promising for applications in tissue repair and regeneration [38,39].

**Scheme 1:**
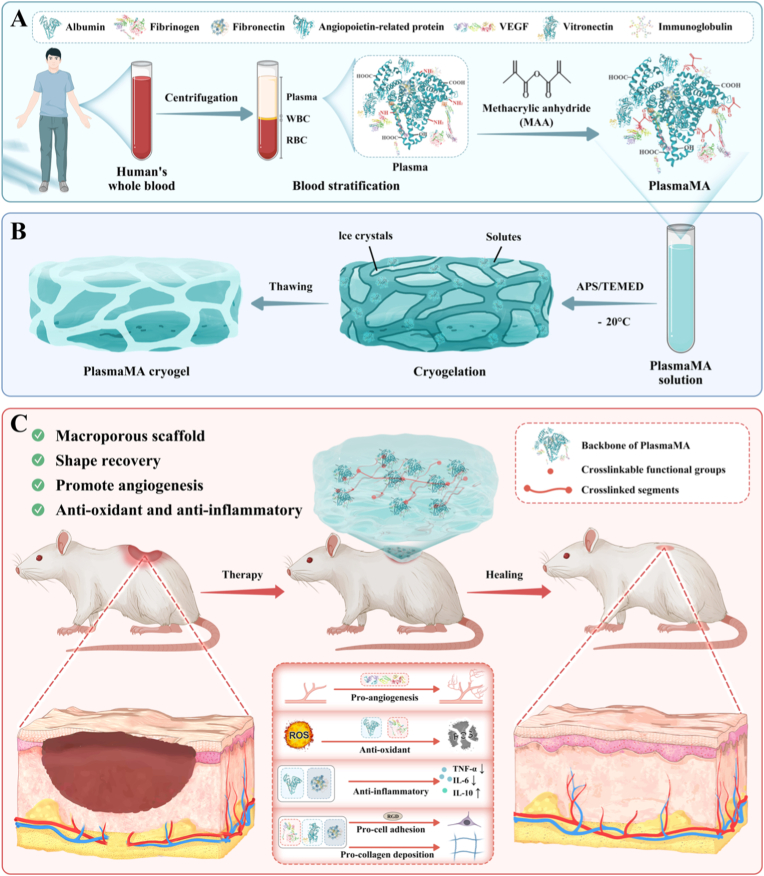
Schematic illustration of the preparation of multifunctional PlasmaMA cryogels for diabetic wound healing. (A) Synthesis of PlasmaMA. (B) Cryogelation of a PlasmaMA solution was induced through the action of APS/TEMED at −20 °C, leading to the formation of the PlasmaMA cryogel. (C) The PlasmaMA cryogel promotes wound healing by exerting multiple biological effects, including angiogenesis, antioxidant activity, anti-inflammatory effect, promotion of cell adhesion and stimulation of collagen deposition.

This study introduces the first development of a human PlasmaMA cryogel scaffold, synthesized by methacrylation of human plasma proteins (Scheme 1A). The resulting scaffold exhibits a highly interconnected macroporous structure (Scheme 1B), enabling efficient nutrient transport and waste clearance at wound sites. Functionally, PlasmaMA cryogels integrate intrinsic multiple bioactive components: VEGF drives angiogenesis and cell migration; albumin and fibrinogen confer antioxidant protection; albumin and fibronectin exert anti-inflammatory effects; fibrinogen, fibronectin and vitronectin promote collagen deposition. Additionally, arginine-glycine-aspartic acid (RGD) motifs present in fibrinogen, fibronectin and vitronectin facilitate ECM remodeling (Scheme 1C). We hypothesize that these multicomponent PlasmaMA cryogel scaffolds create a regenerative microenvironment that recapitulates the native ECM, providing structural support, bioactive signaling, and immune modulation to enhance diabetic wound healing and tissue regeneration.

## 2 Results and discussion

### 2.1 Human PlasmaMA largely retained the secondary structure and protein profile of the original plasma primarily after methacrylation

**Fig. 1:**
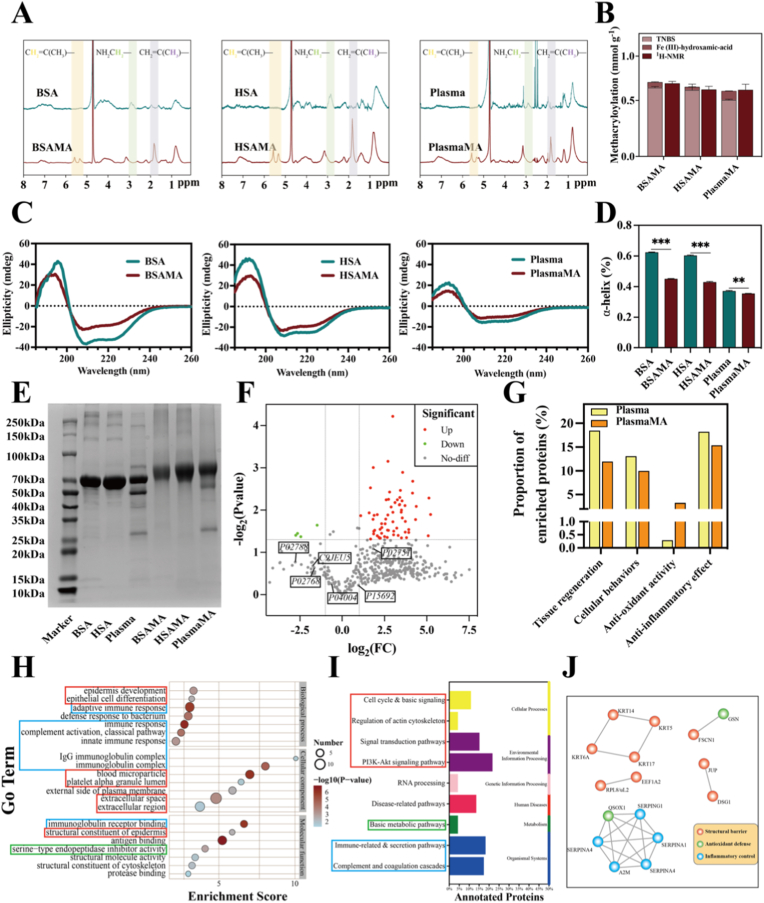
Characterization and biochemical analysis of materials. (A) ^1^H-NMR spectra of BSA, BSAMA, HSA, HSAMA, Plasma, and PlasmaMA. (B) Degree of methacryloyl functionalization from ^1^H-NMR spectra, compared with results of TNBS assay and the Fe(III) hydroxamic acid-based quantification. (C) Circular dichroism (CD) spectra of BSA, BSAMA, HSA, HSAMA, Plasma, and PlasmaMA. (D) α-Helix content derived from CD spectral analysis. (E) SDS-PAGE profiles of BSA, BSAMA, HSA, HSAMA, Plasma, and PlasmaMA, using protein markers used as molecular weight references. (F) Proteomics identification of Plasma and PlasmaMA materials. Volcano plot showing differentially expressed proteins in Plasma and PlasmaMA. (G) Gene Ontology (GO) functional analysis. (H) GO enrichment analysis visualized as a bubble plot. (I) Kyoto Encyclopedia of Genes and Genomes (KEGG) pathway classification of differentially expressed proteins presented as a bar chart. (J) Protein-protein interaction (PPI) network of proteins corresponding to differentially expressed genes. ∗*p*< 0.05, ∗∗*p*< 0.01, and ∗∗∗*p* < 0.001 (n = 3, mean ± SD). ns denotes ‘not significant’.

To confirm successful coupling of methacryloyl groups to BSA, HSA, and Plasma, the original and modified materials were dissolved in D_2_O and analyzed using ^1^H-NMR. As shown in Fig. 1A, new peaks emerged at 5.3–5.6 ppm (C**H_2_**=C(CH_3_)–) and 1.9 ppm (CH_2_=C(C**H_3_**)–), indicating the successful methacrylation of free amino groups present in BSA, HSA, and Plasma. Additionally, the methylene proton peak of the unreacted lysine residue (NH_2_C**H_2_**–) shifted from approximately 2.8 ppm to 3.1 ppm, in BSAMA, HSAMA, and PlasmaMA, further confirming covalent modification [7].

To further validate methacrylation, FT-IR spectroscopy was conducted on the samples, as shown in Fig. S1. In the 3100–3300 cm^−1^ region, the absorption peaks in BSAMA, HSAMA, and PlasmaMA were observed to be weakened and slightly shifted, as compared to their respective unmodified samples. This shift likely results from the reaction of the amino groups (–NH_2_) of the proteins with methacrylic anhydride (MAA), forming amide bonds (–CONH–) and introducing vinyl (–C(CH_3_)=CH_2_) functional groups. These modifications cause a shift in the stretching vibrations of the N–H and sp^2^ C–H bonds. Additionally, an increase in absorption in the 2800–3000 cm^−1^ region suggests the introduction of more sp^3^ C–H bonds, probably originating from the methyl group in the MAA structure. Below 2000 cm^−1^, the intensities of the amide I (∼1650 cm^−1^) and amide II (∼1540 cm^−1^) bands were enhanced, confirming successful amidation. Further confirmation of the incorporation of methacryloyl functional groups was provided by the amplified peaks for C=C stretching (∼1635 cm^−1^) and –CH_3_ bending vibrations (∼1450 cm^−1^). These findings demonstrate that BSA, HSA, and human plasma proteins were successfully incorporated with methacryloyl functional groups.

To quantify the degree of methacrylation, TMSP can be used as an internal reference in the ^1^H-NMR spectrum [40]. To corroborate the methacryloyl quantification obtained via NMR spectroscopy, both TNBS and Fe(III)-hydroxamic acid-based assays were conducted [41]. As shown in Fig. 1B, TNBS assay results revealed methacrylamide contents of 0.69 ± 0.12, 0.67 ± 0.06, and 0.57 ± 0.37 mmol/g for BSAMA, HSAMA, and PlasmaMA, respectively, corresponding to degrees of substitution (DS) of 95.53 ± 0.40%, 94.74 ± 3.64%, and 89.62 ± 0.56% (Fig. S2). Given the three-dimensional shape of BSAMA, HSAMA and PlasmaMA, some amines may be sterically shielded, potentially leading to methacrylation at hydroxyl sites and formation of methacrylate esters [42,43]. The Fe(III)-hydroxamic acid-based assay selectively detects these esterified methacrylate groups, revealing respective contents of 0.06 ± 0.04, 0.03 ± 0.01, and 0.09 ± 0.01 mmol/g in BSAMA, HSAMA, and PlasmaMA. Thus, the combination of TNBS and Fe(III)-hydroxamic acid assays provides a more comprehensive quantification of total methacrylation. Complementary NMR analysis, which directly quantifies the total methacryloyl groups irrespective of attachment to amines or hydroxyls, yielded values of 0.73 ± 0.02, 0.63 ± 0.03, and 0.68 ± 0.06 mmol/g for BSAMA, HSAMA, and PlasmaMA, respectively. Notably, the combined results from the TNBS and Fe(III)-hydroxamic acid-based assays closely approximated the NMR-derived values across all groups, underscoring the reliability and robustness of this integrated quantification strategy [42,43].

To evaluate batch-to-batch consistency, two batches of PlasmaMA samples used in this study were analyzed by ^1^H-NMR, with the degree of methacryloylation calculated using TMSP as an internal standard (Fig. S3). The results showed comparable degrees of methacryloylation between the two batches (0.68 ± 0.06 vs 0.63 ± 0.04, *p* > 0.05), supporting the reproducibility of the methacryloylation process under the current preparation conditions.

The secondary structures of BSA, HSA, Plasma, and their methacrylated derivatives were analyzed by Far-UV Circular Dichroism (CD) spectroscopy [44]. As shown in Fig. 1C, the CD spectra of the proteins before and after methacryloylation exhibit similar trends, suggesting minimal disruption to overall protein conformation. Disulfide bonds (S–S) contribute significantly to the rigidity of albumin subdomains, likely preserving structural stability despite methacryloyl modification. The α-helical content was determined from the characteristic negative band at 208 nm in the CD spectra. As presented in Fig. 1D**,** the α-helix contents of the samples were 62.40 ± 0.24% (BSA), 45.09 ± 0.20% (BSAMA), 60.35 ± 0.35% (HSA), 43.01 ± 0.43% (HSAMA), 37.17 ± 0.33% (Plasma), and 35.51 ± 0.44% (PlasmaMA). The relatively lower α-helical content observed in Plasma compared to pure BSA and HSA is likely attributed to the complex composition of plasma, which contains a diverse array of proteins with varying secondary structures, including β-sheets and random coils. This structural heterogeneity diminishes the dominance of α-helical signatures in CD spectra and reflects the multicomponent nature of plasma-derived biomaterials.

To confirm the diversity of protein components in plasma, we further conducted SDS-PAGE to examine their molecular weight profiles [45]. Fig. 1E reveals that BSA, HSA, and Plasma all exhibit a prominent band at approximately 66 kDa, which corresponds to albumin. Methacryloyl-modified proteins (BSAMA, HSAMA, and PlasmaMA) displayed slightly up-shifted bands relative to their unmodified counterparts, confirming successful methacrylation. The unmodified Plasma sample showed distinct protein bands at approximately 28 kDa, 50 kDa, and 66 kDa. Notably, these characteristic bands remained present in the PlasmaMA sample, indicating that the methacrylation process appeared to preserve the original protein composition. Owing to the mild conditions of methacrylation, reactive sites are typically confined to side chains or regions non-critical to protein structure and function, thereby avoiding direct perturbation of core domains critical to biological functions. Moreover, the MAA-mediated cross-linking proceeds rapidly, limiting the exposure of active molecules to free radicals and reducing the risk of conformational destabilization. Collectively, these features enable methacrylation to achieve efficient cross-linking while possibly retaining the intrinsic bioactivity of proteins.

BSA and HSA are both pure albumin [46,47], whereas human plasma represents a complex mixture of proteins derived from whole blood. To investigate whether methacrylation altered the proteomic profile of plasma, a comparative proteomic analysis was conducted. The volcano plot (Fig. 1F) and quantitative data (Fig. S4) revealed 63 upregulated and 4 downregulated proteins in PlasmaMA relative to native plasma. Key functional proteins, such as albumin (P02768), VEGF (P15692), fibronectin (P02751), fibrinogen (C9JEU5), lactotransferrin (P02788), and vitronectin (P04004), remained abundant after methacrylation, indicating that this modification preserves critical bioactive components. Gene Ontology (GO) analysis revealed that methacrylation retained the core biological functions of plasma, including tissue regeneration, cellular behaviors, antioxidant activity, and anti-inflammatory effect with overall functional integrity well maintained. Notably, GO analysis indicated an enrichment of antioxidant-related proteins, suggesting an enhanced antioxidant capacity following modification (Fig. 1G). GO enrichment analysis (Fig. 1H) showed that differentially expressed proteins were significantly associated with terms such as “extracellular matrix organization [48,49]” and “immune-related processes”, highlighting potential roles in ECM remodeling and immune modulation—both essential for tissue repair and regeneration. The reorganization of the ECM is a critical step in tissue repair, while the enrichment of immune-related terms implies the potential to promote immune homeostasis and accelerate tissue regeneration. Kyoto Encyclopedia of Genes and Genomes (KEGG) pathway analysis (Fig. 1I) further identified significant enrichment in several key signaling pathways related to tissue repair and cellular behavior, including the complement and coagulation cascades, PI3K-Akt signaling, and ECM–receptor interactions. Enrichment of coagulation-related pathways points to a potential role in hemostasis and infection control, while activation of the PI3K-Akt axis may promote cell proliferation, migration, and survival [50]. Enrichment in ECM–receptor interaction pathways underscore the importance of these proteins in mediating cell–matrix adhesion and migration. Protein–protein interaction (PPI) network analysis (Fig. 1J) demonstrated that the upregulated proteins are closely interconnected, forming a compact interaction network. These proteins were predominantly enriched in processes related to immune regulation, antioxidant defense, maintenance of tissue architecture, ECM deposition, and tissue remodeling. These findings indicate that the upregulated proteins may act synergistically to promote tissue regeneration [51]. Proteomic analysis further revealed that multiple plasma-derived proteins remained detectable in PlasmaMA after processing, with functional enrichment associated with tissue repair, antioxidant-related activity, and inflammatory regulation, suggesting partial retention of plasma biofunctional characteristics.

Together, comprehensive proteomic profiling provides mechanistic insight into the multifaceted regenerative capacity of PlasmaMA, involving coordinated regulation of protein synthesis, energy metabolism, ECM remodeling, immune modulation, and signal transduction during tissue repair and regeneration.

### 2.2 Human PlasmaMA cryogels exhibit a macroporous structure, high porosity, tunable biodegradability, excellent mechanical resilience, and the capacity to maintain a moist wound environment

**Fig. 2:**
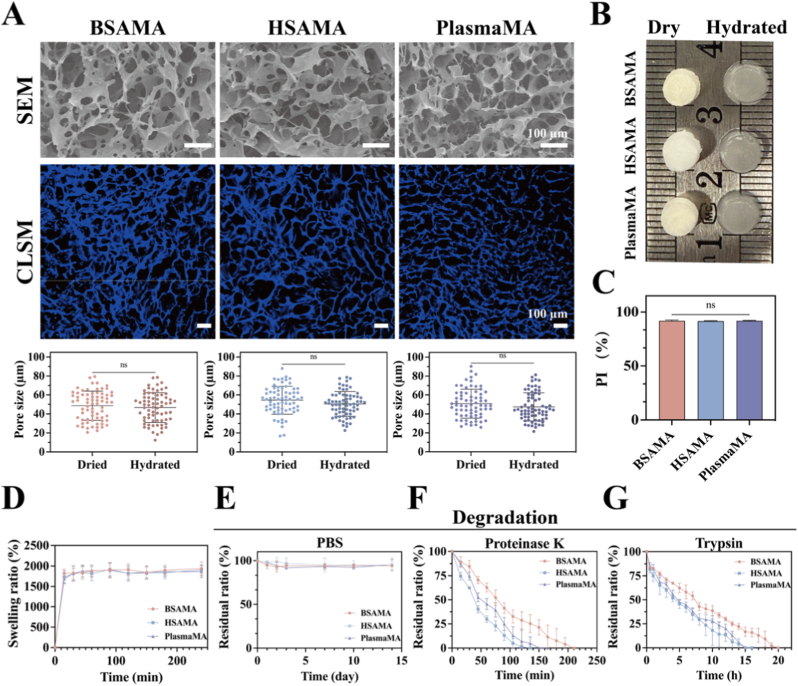
Physicochemical properties of BSAMA, HSAMA, and PlasmaMA cryogels. (A) Morphology analysis of cryogels in dry (SEM) and wet (CLSM) states, along with pore size distribution quantified using ImageJ. (n = 75) (B) Gross appearance of cryogels in dry and wet states. (C) Pore interconnectivity (PI). (D) Swelling behavior. Stability and degradation of cryogels in (E) PBS, (F) 0.01 mg/mL Proteinase K in PBS, and (G) 0.1% Trypsin in PBS. (n = 3, mean ± SD). ns denotes ‘not significant’.

Compared to conventional hydrogels, cryogels exhibit larger pore sizes and higher porosity [52]. Based on initiator and catalyst ratios established in previous studies, we investigated the cryogelation behavior of PlasmaMA at different concentrations [53]. A polymer concentration of 3% was selected as the minimum required for structural integrity and was subsequently used for the preparation of BSAMA, HSAMA, and PlasmaMA cryogels in this study (Table S1). The initiator APS concentration was 0.5% and that of the catalyst TEMED was 0.1% (Table S2). Cryogelation was conducted at −20 °C for 48 h. The morphology of the cryogels was observed using SEM and CLSM. As shown in Fig. 2A, all cryogels presented a well-defined macroporous architecture. SEM images of freeze-dried cryogel samples display pore sizes ranging from 21 to 80 μm for BSAMA, 16 to 87 μm for HSAMA, and 25 to 90 μm for PlasmaMA. The pore size of the swollen cryogels, as observed from CLSM images, was marginally reduced compared to the dried cryogels, with ranges of 12 to 78 μm for BSAMA, 22 to 78 μm for HSAMA, and 21 to 81 μm for PlasmaMA. The macroporous architecture facilitates cell adhesion, migration, and proliferation [54], while also supporting neovascularization, thereby ensuring efficient nutrient delivery, oxygen supply, and waste removal—key factors in enhancing tissue repair and regeneration [55,56]. Notably, both dried and swollen cryogels retained the cylindrical shape defined by the mold, indicating dimensional stability (Fig. 2B).

For application as a biological scaffold, a scaffold should support efficient nutrient diffusion, oxygen permeation, and metabolic waste removal [57]. The highly interconnected porous network of cryogels plays a critical role in fulfilling these requirements. In the present study, all three types of cryogels demonstrated high pore interconnectivity (∼90%), contributing to their large specific surface area and excellent water uptake capacity (Fig. 2C). Swelling ratio analysis demonstrated that all three cryogel groups rapidly reached equilibrium within 30 min. After 24 h of immersion, the swelling ratios of BSAMA, HSAMA, and PlasmaMA cryogels reached 1939.54 ± 165.41%, 1904.82 ± 122.76%, and 1963.96 ± 144.90%, respectively (Fig. 2D).

Moreover, as a candidate biomaterial scaffold for tissue engineering, the degradation behavior of cryogels is of critical importance. Ideally, the degradation rate should align with tissue regeneration to support optimal tissue development [58]. Initially, the stability of these cryogels was evaluated in PBS at 37 °C with shaking (150 rpm). As shown in Fig. 2E, marginal degradation was observed over a period of 14 days (94.20 ± 3.77% for BSAMA cryogels, 95.09 ± 5.16% for HSAMA cryogels, 95.41 ± 6.89% for PlasmaMA cryogels, *p* > 0.05). Corresponding digital images (Fig. S5A) also revealed no noticeable morphological changes during this period. Further, enzyme-mediated degradation was conducted. Proteinase K and Trypsin, both possessing strong proteolytic activity, were employed in these assays. As expected, all cryogels degraded more rapidly in enzymatic environments, with residual mass percentages decreasing significantly over time. In 0.01 mg/mL Proteinase K, the degradation rate of PlasmaMA was intermediate between those of HSAMA and BSAMA. In 0.1% Trypsin, both PlasmaMA and HSAMA fully degraded within approximately 16 h, whereas the degradation of BSAMA required about 20 h (Fig. 2F and G).

To further assess whether the degradation behaviour of PlasmaMA cryogels could be modulated by formulation concentration, PlasmaMA cryogels prepared at 2%, 3%, and 4% were subjected to enzymatic degradation in 0.01 mg/mL Proteinase K and 0.1% Trypsin. As shown in Fig. S6, the degradation kinetics were strongly concentration-dependent under both conditions, with 2% PlasmaMA degrading fastest, 3% PlasmaMA showing an intermediate degradation rate, and 4% PlasmaMA degrading most slowly. Together, these results demonstrate that the degradation profile of PlasmaMA cryogels can be readily tuned by altering the precursor concentration.

To further simulate *in vivo* conditions, long-term co-culture experiments with cells were performed. As shown in Fig. S5B, the cryogels appeared to retain their structural integrity over a 21-day co-culture period. Quantitative analysis revealed that after 35 days of co-culture with HUVECs, the residual ratios of BSAMA, HSAMA, and PlasmaMA cryogels were 45.09 ± 9.06%, 35.24 ± 3.61%, and 47.07 ± 6.32%, respectively. In contrast, co-culture with L929s resulted in slightly higher residual ratios for BSAMA and HSAMA (45.24 ± 5.20% and 37.72 ± 2.02%, respectively), while PlasmaMA showed a lower residual ratio of 36.27 ± 3.76% (Fig. S5C). Based on the above results, PlasmaMA cryogels maintain structural integrity in PBS during the 2-week observation period, while exhibiting accelerated and concentration-dependent degradation under enzymatic conditions, indicating a tunable degradation behavior relevant to tissue engineering applications.

**Fig. 3:**
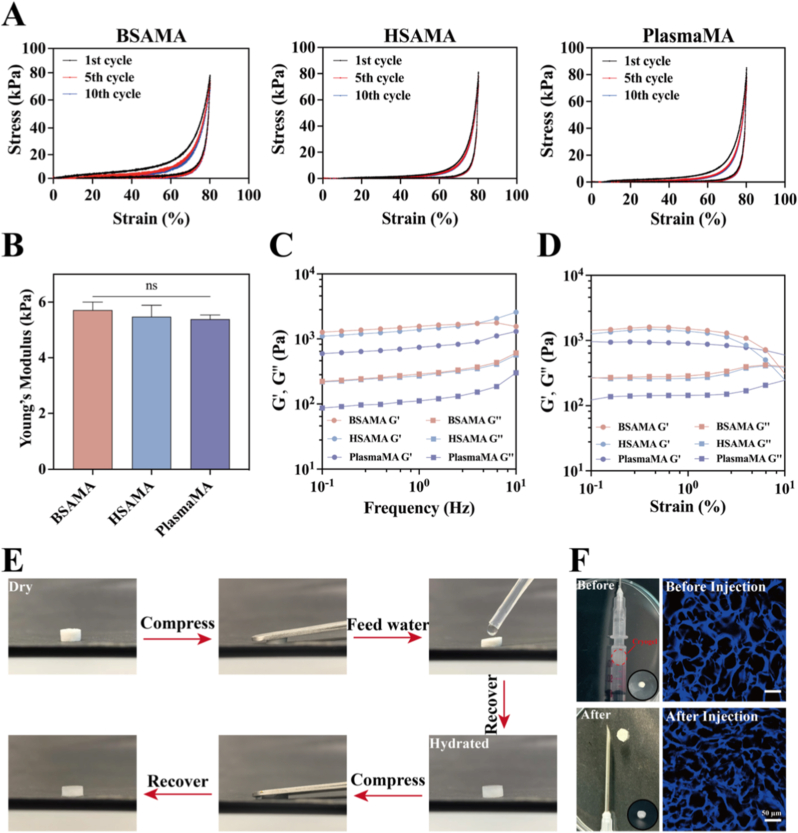
Mechanical characterization of BSAMA, HSAMA, and PlasmaMA cryogels. (A) Cyclic compression behavior of the three cryogels over 10 cycles at 80% strain. (B) Young's modulus calculated from the slope of the stress–strain curve within the linear elastic region (0–20% strain). (C) Frequency sweep curves obtained at 0.2% strain. (D) Storage modulus (G′) and loss modulus (G″) as functions of strain (0.1–10%) at 1 Hz. (E) Shape recovery ability of PlasmaMA cryogels and (F) Digital photographs demonstrate the pre-injection loading of PlasmaMA cryogel into a 1-mL syringe and its post-injection recovery through a standard 16-gauge needle, demonstrating its injectability. Confocal microscopy images reveal the porous microarchitecture of the PlasmaMA cryogel before and after injection, with DAPI staining delineating the scaffold matrix. (n = 3, mean ± SD); ns denotes ‘not significant’.

As a scaffold material, the mechanical properties of cryogels are pivotal for supporting cell growth and migration. Adequate mechanical properties not only offer stable structural support for cellular adhesion and proliferation but also facilitate cell infiltration [59]. Moreover, scaffolds with sufficient mechanical integrity can bear the developing tissue load and sustain the overlying epidermal architecture, thereby providing essential physical cues and a conducive mechanical microenvironment for effective tissue regeneration [60]. To evaluate the mechanical properties of the cryogels, cyclic compression tests were conducted on BSAMA, HSAMA, and PlasmaMA cryogels. As depicted in Fig. 3A, all three cryogels retained their structural integrity over 10 successive loading–unloading cycles at an 80% strain level, indicating pronounced mechanical resilience. Young's modulus, determined from the linear region (10–20% strain) of the stress–strain curves (Fig. 3B), was calculated as 5.6 ± 0.28 kPa for BSAMA, 5.4 ± 0.41 kPa for HSAMA, and 5.3 ± 0.15 kPa for PlasmaMA. Additionally, dynamic frequency sweep analyses (Fig. 3C and D) revealed that across a frequency range of 0.1 to 10 Hz, the storage modulus (G′) consistently exceeded the loss modulus (G″), indicative of solid-like elastic behavior. Strain amplitude sweeps (at 1 Hz, ranging from 0.1% to 10%) further confirmed the dominance of G′ over G″, highlighting the cryogels' excellent shear resistance and viscoelastic stability [61]. Collectively, cryogels exhibit suitable mechanical properties and integrity, ensuring sufficient biomechanical support for cell growth, migration, and tissue regeneration.

As demonstrated in Fig. 3E, the lyophilized PlasmaMA cryogel rapidly regained its original shape upon rehydration, even after being subjected to strong compression. The pronounced shape-recovery behavior of the cryogel underscores its exceptional structural elasticity, enabling swift recovery from mechanical deformation while preserving the internal architecture crucial for sustained cell migration, proliferation, and efficient tissue regeneration [62]. We evaluated the feasibility of delivering PlasmaMA cryogels via a conventional needle and assessed their post-injection structural recovery. Under injection-induced shear stress, the gels underwent transient volume reduction due to temporary polymer network collapse [63]. However, owing to their high compressive tolerance and intrinsic shape-recovery properties, the cryogels rapidly returned to their original shape without permanent deformation or mechanical damage upon release of the applied force. PlasmaMA cryogels (4 mm × 4 mm × 1 mm) suspended in 0.5 mL PBS were successfully injected through a 16-gauge needle (Video S1), maintaining their macroscopic integrity post-extrusion. To assess preservation of microarchitecture, cryogels were stained with DAPI before and after injection and examined by confocal microscopy. No significant differences in pore morphology or pore size before and after injection were observed (*p* > 0.05), confirming that the 3D porous structure remained intact during injection (Fig. 3F and Fig. S7).

Supplementary data related to this article can be found online at https://doi.org/10.1016/j.mtbio.2026.103151

The following are the Supplementary data related to this article:

Video

Overall, the developed PlasmaMA cryogels possess an interconnected macroporous structure, high porosity, appropriate biodegradability, excellent mechanical properties, and swelling capability (for maintaining a moist wound environment), making them promising candidates for applications in wound healing and tissue regeneration.

### 2.3 Human PlasmaMA cryogels promoted cell adhesion, proliferation, migration, sustained VEGF release, angiogenesis, and antioxidant activity *in vitro*

In the context of tissue engineering and regenerative medicine, effective cell adhesion enables the biomaterial to mimic the native ECM microenvironment and modulate various cellular behaviors, including proliferation, migration, and differentiation [64,65]. Human plasma is inherently rich in extracellular matrix proteins such as fibronectin, fibrinogen, and vitronectin, which contain integrin-recognizable peptide sequences (e.g., RGD motifs). These sequences can specifically bind to integrin receptors on the cell surface, thereby promoting firm and stable adhesion. Moreover, cryogel-based scaffolds typically possess a highly porous and interconnected microarchitecture, which further facilitates cellular infiltration and adhesion [66,67].

**Fig. 4:**
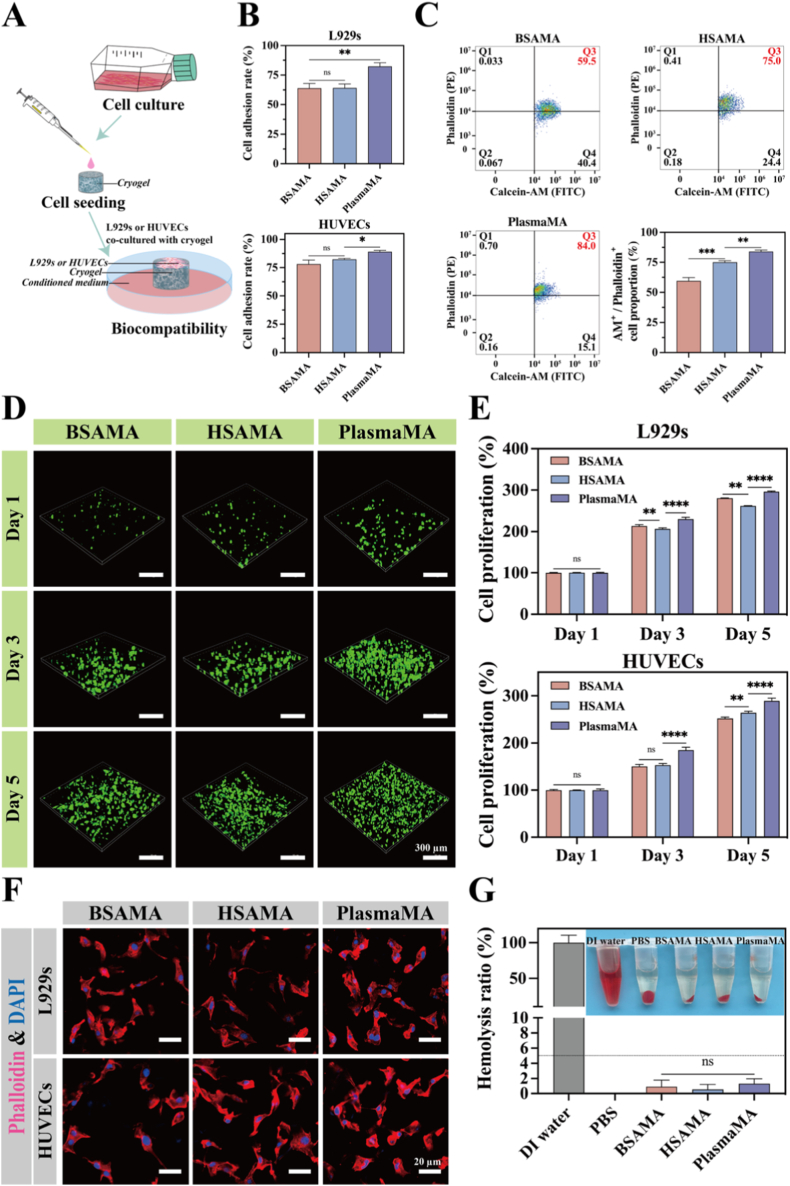
Cell compatibility and proliferation on BSAMA, HSAMA, and PlasmaMA cryogels. (A) Schematic overview of the experimental design to evaluate cell compatibility and proliferation on the surface of BSAMA, HSAMA, and PlasmaMA cryogels. (B) Cell adhesion rate of L929s and HUVECs, assessed 2.5 h after 3D cell seeding. (C) Flow cytometry analysis of HUVECs stained with Calcein-AM and Rhodamine-Phalloidin after 4 h of culture on cryogels, indicating cell viability and F-actin content, respectively. (D) Live/Dead staining of L929s co-cultured with cryogels over 1, 3, and 5 days. (E) Cell proliferation of L929s and HUVECs co-cultured with cryogels over 1, 3, and 5 days. (F) Immunofluorescence staining of L929s and HUVECs after 5 days of co-culture with cryogels, visualized using CLSM (DAPI: blue; phalloidin: red). Scale bar: 20 μm. (G) Hemolysis rate of cryogels indicating their hemocompatibility. ∗*p*< 0.05, ∗∗*p*< 0.01, ∗∗∗*p*< 0.001, and ∗∗∗∗*p* < 0.0001 (n = 3, mean ± SD); ns denotes ‘not significant’. (For interpretation of the references to color in this figure legend, the reader is referred to the Web version of this article.)

To evaluate the cell adhesion capacity of the cryogels, adhesion rates were measured as shown in Fig. 4A and B. PlasmaMA cryogels supported the highest cell adhesion rates among the groups. For L929s, the adhesion rates on BSAMA, HSAMA, and PlasmaMA cryogels were 63.97 ± 3.95%, 64.25 ± 3.32%, and 82.33 ± 3.08%, respectively; for HUVECs, the corresponding values were 78.09 ± 3.63%, 82.32 ± 0.94%, and 89.13 ± 1.25%, respectively. These results demonstrate the superior cell adhesion capacity of PlasmaMA across both fibroblast and endothelial cell types.

To further assess the early adhesion-promoting capacity of PlasmaMA cryogels, HUVECs were seeded onto three types of cryogel scaffolds for 4 h (Fig. 4C). Flow cytometry with Calcein-AM and Rhodamine-Phalloidin staining was used to quantify adherent cells and evaluate cytoskeletal organization. Phalloidin selectively binds to filamentous actin (F-actin), and its fluorescence intensity reflects the extent of cytoskeletal remodeling. Thus, quantitative analysis of F-actin fluorescence serves as a reliable indicator of cytoskeletal organization. After 4 h of incubation, cells in all three groups had largely adhered to the scaffold surfaces. Calcein-AM staining indicated comparable cell viability (above 99%) among the groups, whereas Phalloidin staining revealed markedly enhanced F-actin expression in the PlasmaMA group, suggesting more pronounced cytoskeletal remodeling. The proportion of Calcein-AM^+^/Phalloidin^+^ cells in the PlasmaMA (84.07 ± 1.27%) group was significantly higher than in the BSAMA (59.53 ± 2.76%, *p* < 0.001) and HSAMA (75.03 ± 1.72%, *p* < 0.01) groups. As shown in Fig. S8, flow cytometric analysis of L929s adhesion revealed double-positive (Calcein-AM^+^/Phalloidin^+^) proportions of 62.4 ± 0.70%, 67.9 ± 1.86%, and 74.3 ± 4.58% in the BSAMA, HSAMA, and PlasmaMA groups, respectively, which is consistent with the trend observed in HUVECs. These results indicate that PlasmaMA not only facilitates early cell adhesion but also significantly promotes cytoskeletal reorganization. This effect may be attributed to the presence of extracellular matrix proteins in PlasmaMA—such as fibronectin, fibrinogen, and vitronectin—which contain RGD motifs capable of binding integrins (e.g., β1 and αvβ3), thereby activating downstream signaling pathways involved in adhesion and F-actin organization. CD29 (integrin β1) is broadly expressed on the surface of various cell types and plays a central role in mediating cell–ECM adhesion and downstream signal transduction [68,69]. In the CD29-blocking assay, HUVECs in the untreated and CD29-blocked groups were stained with Calcein-AM, and fluorescence intensity was quantitatively analyzed by flow cytometry (Fig. S9). The CD29-blocked group exhibited a significantly reduced Calcein-AM fluorescence signal compared to the untreated group (*p* < 0.0001), indicating a marked decrease in cell adhesion capacity. These results suggest that the PlasmaMA cryogel surface contains ligands capable of engaging integrin β1, facilitating cell attachment via this pathway. Notably, a residual level of adhesion persisted even after integrin β1 blockade, implying the involvement of additional adhesion mechanisms beyond the β1 integrin axis.

L929s and HUVECs were seeded separately onto the cryogel surfaces, and their viability, proliferation, and morphology were subsequently evaluated over time. As shown in Fig. 4D, L929s were cultured on the cryogels for 1, 3, and 5 days with cell viability assessed using the Calcein-AM/EthD-1 (Live/Dead) assay. The majority of cells in all three groups exhibited green fluorescence (Calcein-AM), with minimal red fluorescence (EthD-1), indicating high viability across all time points. A progressive increase in green fluorescence intensity and cell confluence was observed over time, suggesting sustained proliferation. Among the tested groups, the PlasmaMA cryogels supported the highest cell density, indicating enhanced cellular attachment and growth.

To further assess cell proliferation, the CCK-8 assay was employed to evaluate the metabolic activity of L929s and HUVECs co-cultured with the cryogels. As shown in Fig. 4E, both cell types exhibited higher proliferation on PlasmaMA cryogels compared to BSAMA and HSAMA groups over the 5-day period. On day 5, the relative proliferation (normalized to day 1) of L929s reached 280.26 ± 6.74% (*p* < 0.0001) on BSAMA cryogels, 261.73 ± 9.04% (*p* < 0.0001) on HSAMA cryogels, and 294.11 ± 12.96% on PlasmaMA cryogels. For HUVECS, the relative proliferation values were 250.03 ± 3.07% (*p* < 0.0001) on BSAMA cryogels, 264.10 ± 3.09% (*p* < 0.0001) on HSAMA cryogels, and 289.32 ± 5.75% on PlasmaMA cryogels. These results demonstrate that the PlasmaMA cryogel significantly enhances cellular proliferation compared to the other albumin cryogels, highlighting its superior cell adhesion and pro-proliferative properties.

To evaluate cellular compatibility, cells were exposed for 24 h to extracts from the PlasmaMA cryogel and from the corresponding unpolymerized PlasmaMA precursor at identical concentrations. Importantly, cells treated with the PlasmaMA cryogels exhibited significantly higher viability than those treated with the corresponding PlasmaMA precursor (Fig. S10). Both L929s and HUVECs exhibited higher viability in the cryogel extract group than in the precursor group. Interestingly, cell viability was significantly greater with cryogel extracts than with precursor solutions. These results indicate that the cryogelation process itself does not introduce appreciable cytotoxicity.

F-actin is a key component of the cytoskeleton and is primarily localized along the cell periphery and within lamellipodia—structures associated with cell adhesion and motility [70]. To assess cell morphology and spreading, active filaments were labeled with Rhodamine-Phalloidin. As shown in Fig. 4F, both L929s and HUVECs exhibited a characteristic spindle-shaped morphology on all three cryogels, with organized F-actin bundles and extended cellular processes. These results further confirm that all three cryogels support cell attachment and cell spreading.

Before the clinical application of biological scaffolds, it is crucial to assess their hemocompatibility, particularly their potential to induce hemolysis. Excessive hemolysis can cause erythrocyte lysis and hemoglobin release, impairing oxygen transport and potentially leading to tissue damage, which could hinder tissue regeneration and compromise scaffold safety [69]. As shown in Fig. 4G, the DI water group exhibited marked hemolysis, whereas the BSAMA, HSAMA, and PlasmaMA groups all maintained hemolysis ratios below 5% [71]. These results demonstrate their excellent hemocompatibility and minimal risk of inducing red blood cell lysis, underscoring their suitability for applications involving direct blood contact.

Growth factors are critical regulators of tissue repair and regeneration, modulating key cellular behaviors such as proliferation, migration, and differentiation [13,72]. These bioactive molecules shape the local microenvironment and expedite functional restoration of damaged tissues. However, conventional strategies that rely on the delivery of a single recombinant growth factor—such as VEGF or FGF—are limited in their ability to mimic the complex, dynamic signaling environment of the native ECM [73]. In addition, these approaches lack the synergistic roles of adhesion ligands, structural proteins, and essential nutrients that together constitute a physiologically supportive microenvironment [74]. Additionally, conventional delivery systems often exhibit burst release and poor bioactivity retention, limiting their effectiveness in tissue regeneration [75,76].

PlasmaMA, a biomaterial derived from human plasma, is intrinsically enriched with a broad spectrum of endogenous growth factors—including VEGF and epidermal growth factor (EGF)—as well as cytokines, nutritional elements, and matrix proteins. This molecular complexity offers a robust foundation for establishing a bioactive microenvironment that closely mirrors native tissue conditions.

**Fig. 5:**
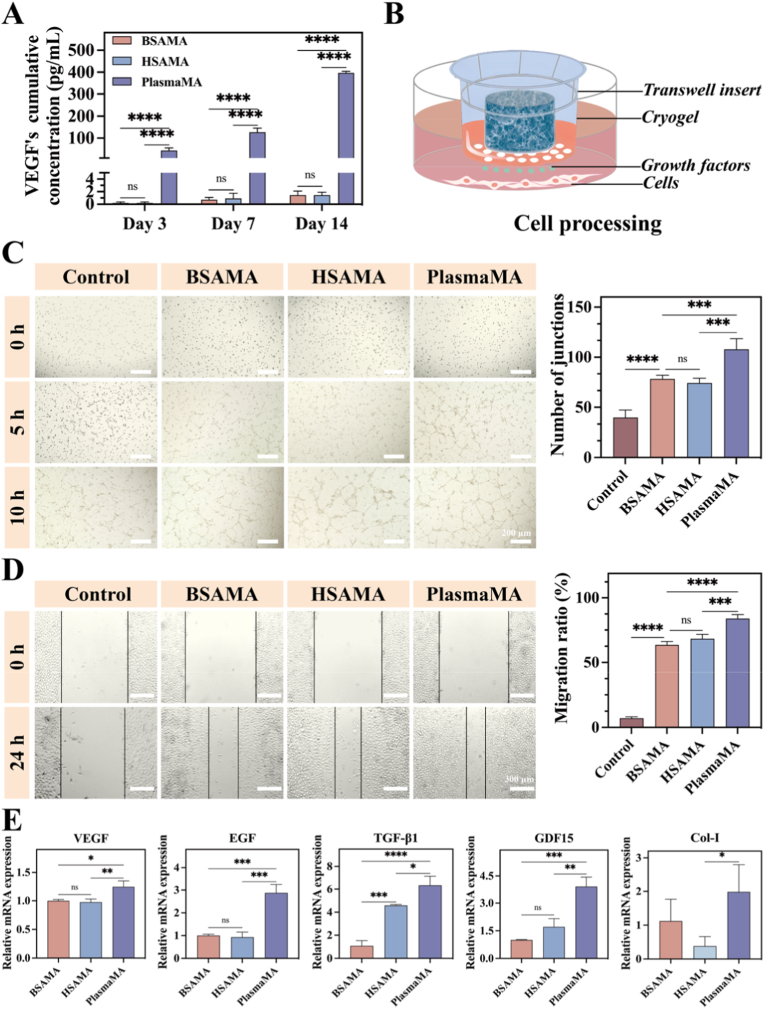
Assessment of angiogenesis and cell migration of BSAMA, HSAMA, and PlasmaMA cryogels. (A) ELISA quantification of VEGF release from different cryogels in PBS extracts. (B) Schematic diagram of the transwell experimental setup used to assess cell migration. (C) Representative images from *in vitro* angiogenesis assays at 0, 5, and 10 h, along with quantitative analysis of tube branching points at 10 h. Scale bar: 200 μm. (D) Migration behavior of L929s in scratch wound assays at 0 and 24 h, with quantitative assessment of scratch closure at 24 h. Scale bar: 300 μm. (E) Relative gene expression levels of VEGF, EGF, TGF-β1, GDF-15, and Col-I in HUVECs and L929s following 4 days of co-culture with cryogels. ∗*p*< 0.05, ∗∗*p*< 0.01, ∗∗∗*p*< 0.001, and ∗∗∗∗*p* < 0.0001 (n = 3, mean ± SD); ns denotes ‘not significant’.

As a key regulator for angiogenesis, VEGF plays a central role in tissue repair. Given the pivotal role of angiogenesis in tissue repair, VEGF was employed as a representative marker and its release from the cryogels was quantified over time using enzyme-linked immunosorbent assay (ELISA). The standard curve for VEGF detection is provided in Fig. S11. The PlasmaMA cryogels exhibited a sustained release of VEGF over a 14-day period as illustrated in Fig. 5A. The cumulative release of VEGF reached 44.12 ± 11.66 pg/mL by day 3, 126.9 ± 18.88 pg/mL by day 7, and 386.3 ± 6.56 pg/mL by day 14. In contrast, VEGF levels in the BSAMA and HSAMA groups remained negligible (∼1.45 ± 0.65 pg/mL and 1.45 ± 0.47 pg/mL, respectively, *p* < 0.0001 vs. PlasmaMA), with no significant increase over time. These findings suggest that PlasmaMA contains bioactive, endogenous VEGF derived from plasma, which is gradually released. This sustained delivery profile highlights the potential of PlasmaMA as an intrinsic pro-angiogenic scaffold.

Cell migration and proliferation are essential during tissue regeneration [77]. To evaluate the angiogenic potential of PlasmaMA cryogels on HUVECs and their migratory effect on L929s, angiogenesis experiments were conducted using the cryogel extracts for HUVECs. For L929s, a Transwell-based scratch assay was employed, as shown in Fig. 5B.

As shown in Fig. 5C, the proangiogenic potential of BSAMA, HSAMA, and PlasmaMA cryogel extracts was evaluated using an *in vitro* tube formation assay with HUVECs cultured on Matrigel. After 5 h of incubation, cells in all cryogel-treated groups began to form vascular-like structures. Angiogenesis was quantified by counting the number of branch points, which reflects the complexity of the vascular network—more branch points indicate more active and organized vascularization. By 10 h, the PlasmaMA group exhibited the most extensive network formation, with 107 ± 7.81 branch points—significantly higher than BSAMA (78 ± 3.50, *p* < 0.001) and HSAMA (74 ± 4.57, *p* < 0.001). All three cryogel extract-treated groups showed markedly enhanced angiogenesis, as compared to the Control group (40 ± 7.54 branch points, *p* < 0.0001). The robust angiogenic response is likely attributed to the presence of albumin, which has been reported to promote endothelial cell proliferation and migration and have antioxidant and anti-inflammatory effects that improve the regenerative microenvironment [78,79]. Notably, the superior performance of PlasmaMA is further supported by its endogenous VEGF content, as demonstrated in the sustained release profile (Fig. 5A) [80,81]. Albumin may bind and stabilize growth factors like VEGF, prolonging their action and enhancing their function. Together, these findings highlight the superior proangiogenic capacity of PlasmaMA cryogels, driven by synergistic contributions of albumin and intrinsic growth factors.

To assess the migratory capacity of fibroblasts, scratch wound healing assays were performed using L929s treated with cryogel extracts. As shown in Figs. 5D, 24 h after wounding, the PlasmaMA group exhibited the greatest reduction in scratch width, indicating enhanced cell migration. The PlasmaMA group had a faster wound closure rate of 83.94 ± 6.61% than the Control group (7.09 ± 1.08%, *p* < 0.0001), BSAMA group (63.50 ± 2.62%, *p* < 0.0001), and HSAMA group (68.23 ± 3.40%, *p* < 0.001). These results demonstrate that PlasmaMA cryogels most effectively promote fibroblast migration, a critical process in wound healing and tissue repair. Collectively, the data show that PlasmaMA cryogels support sustained VEGF release, enhancing angiogenesis in HUVECs and promoting fibroblast migration. This dual pro-regenerative activity underscores the potential of PlasmaMA as a bioactive scaffold for tissue engineering.

To elucidate the molecular mechanisms underlying the regenerative effects of the cryogels, we analyzed the expression of key genes involved in angiogenesis, proliferation, and ECM remodeling in both HUVECs and L929s. VEGF [82] and EGF [83] play important roles in angiogenesis and cell proliferation. Transforming growth factor-β1 (TGF-β1) promotes cell proliferation and aids in tissue repair [84]. The expression of growth differentiation factor-15 (GDF-15) increases in response to tissue damage [85], contributing to the recovery of tissue homeostasis by regulating cellular stress and inflammatory responses. Col-I, a key protein secreted by fibroblast in the ECM, is critical for tissue structure and repair [86]. HUVECs were co-cultured with BSAMA, HSAMA, and PlasmaMA cryogels for 4 days to assess the relative expression levels of genes such as VEGF, EGF, TGF-β1, and GDF-15. In HUVECs, qRT-PCR analysis showed that PlasmaMA cryogels exhibited the highest expression levels of VEGF, EGF, TGF-β1, and GDF-15 compared to the other groups. Similarly, L929s were co-cultured with BSAMA, HSAMA, and PlasmaMA cryogels for 4 days to assess the relative expression of the Col-I gene(Fig. 5E). Compared to the HSAMA group, the PlasmaMA group exhibited significantly higher Col-I expression (*p* < 0.05). This suggests that the PlasmaMA cryogel not only enhances angiogenesis through upregulation of VEGF and EGF but also supports tissue remodeling and repair through increased collagen synthesis and regulation of inflammatory responses.

HGF and TGF-α are key regulators of cell proliferation and migration, playing pivotal roles in tissue repair and regeneration [87]. BMPR2 contributes to cellular differentiation and modulates angiogenesis and migration [88]. Meanwhile, Col-IIα1, is essential for maintaining structural stability within tissues, underscoring its importance in regenerative matrix integrity [89]. The expression levels of HGF, TGF-α, BMPR2, and Col-IIα1 were markedly elevated in the PlasmaMA group compared to the BSAMA and HSAMA groups (Fig. S12A–D). Notably, Col-IIα1 expression was the most significantly upregulated in the PlasmaMA group compared to both BSAMA and HSAMA (*p* < 0.0001), suggesting a superior capacity to promote ECM synthesis and structural tissue reconstruction. Concurrently, the increased expression of HGF and TGF-α in the PlasmaMA group suggests enhanced proliferative and migratory signaling, while the upregulation of BMPR2 in the PlasmaMA group may play a role in matrix remodeling. Collectively, these results demonstrate that the PlasmaMA cryogel activates multiple regenerative pathways at the transcriptional level—promoting angiogenesis, fibroblast activity, collagen production, and stress response regulation. This multifaceted molecular response underscores its potential as a bioactive scaffold for tissue repair.

The implantation of biomaterials often triggers an immune response, leading to the production of reactive oxygen species (ROS), which can cause oxidative damage to lipids, proteins, and DNA, ultimately impairing tissue regeneration [90]. Therefore, evaluating the antioxidant capacity of biomaterials is important for assessing their potential biocompatibility and therapeutic performance. Plasma contains a variety of proteins that have been reported to participate in redox regulation. For example, albumin, particularly through its free thiol groups and cysteine residues, has been implicated in ROS scavenging and redox buffering, and its antioxidant role has also been linked to its ability to bind redox-active metal ions [91]. Fibrinogen has also been reported in the context of ROS-associated structural and functional regulation, suggesting a potential role in oxidative-stress-related biological responses [92]. Lactotransferrin (lactoferrin) has been implicated in antioxidant regulation, particularly through iron-binding activity that may limit iron-driven ROS generation and has also been associated with the modulation of cellular antioxidant pathways such as Sesn2/Nrf2 in lactoferrin-containing systems [93,94]. On this basis, the antioxidant-related performance of PlasmaMA cryogels may be related, at least in part, to the complex plasma-derived protein milieu retained within the material. However, the precise molecular mechanism underlying this effect was not investigated in the present study and requires further clarification.

**Fig. 6:**
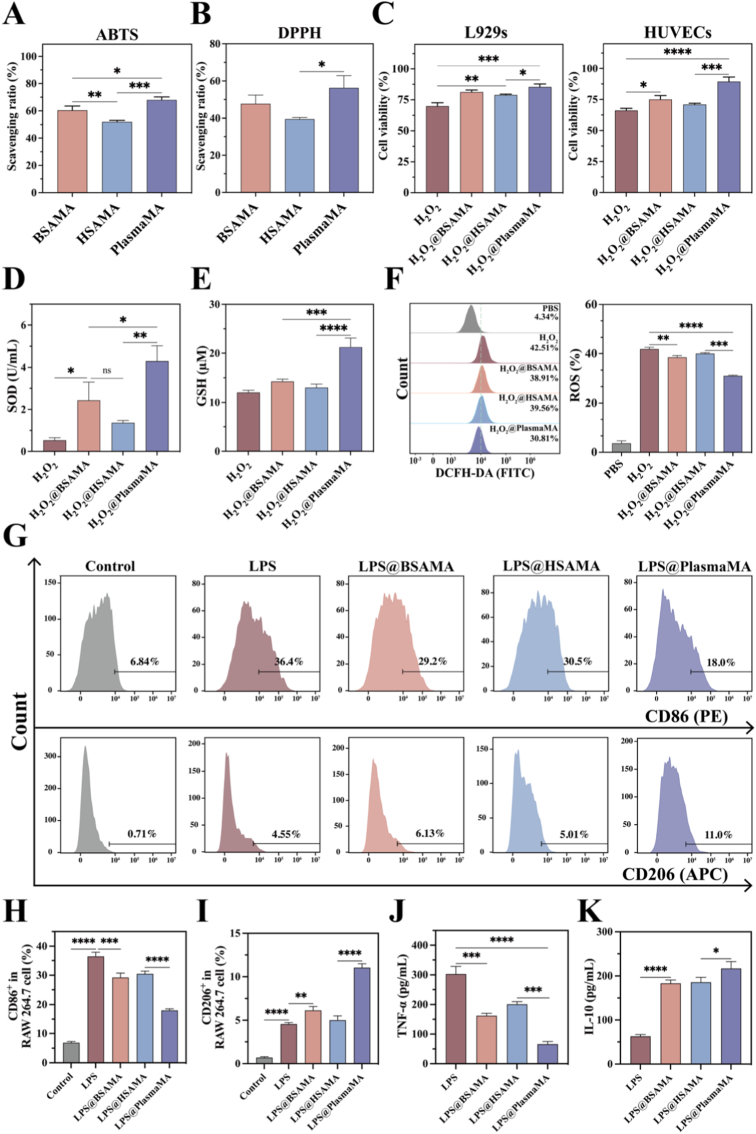
Antioxidant and anti-inflammatory properties of BSAMA, HSAMA, and PlasmaMA cryogels. (A) ABTS and (B) DPPH radical scavenging ability of BSAMA, HSAMA, and PlasmaMA cryogels. L929s and HUVECs were exposed to 1 mM H_2_O_2_ and subsequently cultured with DMEM extracts derived from BSAMA, HSAMA, or PlasmaMA cryogels. (C) Cell viability following treatment with cryogel extracts. (D) SOD activity and (E) intracellular GSH level in L929s under H_2_O_2_ stimuli. (F) Flow cytometry quantification of ROS-positive cells by DCFH-DA staining. (G–I) Flow cytometry analysis of CD86 and CD206 expression on RAW 264.7 macrophages and corresponding quantification. (J, K) ELISA detection of TNF-α and IL-10 levels in LPS-stimulated RAW 264.7 cells. ∗*p*< 0.05, ∗∗*p*< 0.01, ∗∗∗*p*< 0.001, and ∗∗∗∗*p* < 0.0001 (n = 3, mean ± SD); ns denotes ‘not significant’.

The antioxidant activities of BSAMA, HSAMA, and PlasmaMA cryogels were evaluated through ABTS and DPPH scavenging assays. As shown in Fig. 6A and B, all three cryogels demonstrated notable radical scavenging activity. Among them, PlasmaMA cryogels exhibited the strongest antioxidant effect, with an ABTS radical scavenging rate of 68.04 ± 2.29% and a DPPH radical scavenging rate of 56.24 ± 6.60%. In the ABTS assay, BSAMA showed a scavenging rate of 60.47 ± 3.01%, while HSAMA exhibited a rate of 52.11 ± 4.11%. In the DPPH assay, the scavenging rates for BSAMA and HSAMA were 47.67 ± 4.75% and 39.56 ± 0.85%, respectively. The corresponding color changes were visually observed during the assay, as presented in Fig. S13. These results demonstrate that PlasmaMA cryogels possess superior radical scavenging activity compared with BSAMA and HSAMA. This enhanced antioxidant-related performance may be associated with the more complex bioactive protein composition of plasma.

H_2_O_2_ at high concentrations could induce oxidative stress, leading to damage of key cellular components including membranes, proteins, and DNA. Cell viability of L929s and HUVECs under 1 mM H_2_O_2_ stimulation was quantitatively assessed using the CCK-8 assay. As shown in Fig. 6C, the cell viability of L929s in the PlasmaMA group reached 85.45 ± 3.26%, which was significantly higher than that in the H_2_O_2_ group (69.77 ± 2.67%, *p* < 0.001), as well as in the BSAMA (81.21 ± 3.71%, *p* < 0.05) and HSAMA (78.92 ± 2.79%, *p* < 0.05) groups. Meanwhile, the viability of HUVECs in the PlasmaMA group was 89.39 ± 3.61%, which is higher than that of H_2_O_2_ group (66.11 ± 1.61%, *p* < 0.0001), BSAMA group (74.98 ± 3.10%, *p* < 0.001), and HSAMA group (70.77 ± 2.81%, *p* < 0.001).

After 24 h of incubation with cryogel-treated H_2_O_2_ solutions, biochemical assays for SOD and GSH were conducted to evaluate oxidative stress responses. Upon H_2_O_2_ stimulation, SOD acts as the first line of defense by eliminating superoxide anion radicals (O_2_^•-^) [95]. Subsequently, GSH, primarily through the action of glutathione peroxidase (GPx), participates in the reduction of H_2_O_2_ and other reactive oxygen species (ROS), thereby maintaining redox homeostasis [96]. As shown in Fig. 6D and E, the SOD assay revealed a marked increase in SOD activity in all scaffold-treated groups compared to the H_2_O_2_ group (0.52 ± 0.12 U/mL). Specifically, the BSAMA, HSAMA, and PlasmaMA groups exhibited SOD levels of 2.44 ± 0.86 U/mL, 1.36 ± 0.61 U/mL, and 4.30 ± 0.72 U/mL, respectively, indicating that all three cryogels enhanced cellular antioxidant defense under oxidative stress conditions, with PlasmaMA showing the most pronounced effect. In addition, the GSH assay demonstrated increased intracellular glutathione levels in the scaffold-treated groups relative to the H_2_O_2_ Control (12.03 ± 0.44 μM). The BSAMA and HSAMA groups reached 14.27 ± 0.46 μM and 13.03 ± 0.70 μM, respectively, while the PlasmaMA group exhibited the highest level of 21.27 ± 1.88 μM among the groups, suggesting its superior capacity to enhance cellular antioxidant defenses.

To visualize intracellular ROS levels, L929s were stained with DCFH-DA fluorescent dye, analyzed via flow cytometry and CLSM. Flow cytometry results (Fig. 6F) revealed that the ROS level in the H_2_O_2_-treated positive Control group was 42.51 ± 3.85%(*p* < 0.0001), whereas PlasmaMA-treated cells showed a significantly reduced ROS level of 30.81 ± 2.77%, lower than the levels observed in the BSAMA-treated group (38.91 ± 2.74%, *p* < 0.001) and HSAMA-treated group (39.56 ± 2.39%, *p* < 0.001). CLSM images corroborated the flow cytometry findings. The H_2_O_2_-treated positive Control group exhibited the highest fluorescence intensity, whereas the other three treatment groups showed progressively reduced fluorescence levels, as illustrated in Fig. S14. Quantitative fluorescence analysis demonstrated that BSAMA- (*p* < 0.05) and HSAMA-treated (*p* < 0.05) groups moderately attenuated intracellular ROS levels relative to the H_2_O_2_ group. In contrast, the PlasmaMA-treated group exhibited a pronounced reduction in ROS accumulation, lower than both BSAMA (*p* < 0.01) and HSAMA (*p* < 0.001) groups, highlighting its superior ROS-scavenging capability and enhanced cytoprotective effect against oxidative stress. Collectively, these results demonstrate that PlasmaMA cryogels effectively attenuated oxidative stress and provided stronger cytoprotection than BSAMA and HSAMA under H_2_O_2_-induced conditions.

Macrophage phenotype is typically defined by the expression of surface markers such as CD86 (M1) and CD206 (M2) [97]. In diabetic wounds, a sustained pro-inflammatory microenvironment hinders the transition of macrophages from the M1 to the reparative M2 phenotype, thereby exacerbating inflammation and impairing tissue regeneration [98,99]. Vicente Arroyo et al. demonstrated that albumin bound to LPS and inhibited Toll-like receptor signaling, thereby downregulating proinflammatory cytokines such as TNF-α and suppressing M1 macrophage polarization, underscoring its immunomodulatory potential [100]. Similarly, Javier Fernández et al. reported that albumin scavenged ROS, transported inflammatory mediators, and improved the tissue microenvironment, alleviating inflammation through multiple pathways [101]. Collectively, these findings indicate that albumin exerts multifaceted immunomodulatory effects capable of inhibiting sustained M1 polarization of macrophages. Katja Pietsch et al. found that functionalized fibronectin induced a non-classical, immune-tolerant macrophage phenotype. Under steady-state conditions, it suppressed pro-inflammatory cytokines like IL-6 and CXCL10, sustained IL-10 expression, and preserved inflammatory responsiveness to LPS, indicating reversible immunomodulatory effects [102]. Based on this, we hypothesized that PlasmaMA containing albumin and fibronectin may synergistically exert both anti-inflammatory and pro-regenerative effects.

Immune regulation plays a pivotal role in tissue injury and repair by eliminating pathogens and cellular debris while curbing excessive inflammation that can lead to secondary damage [103]. Central to this process are M2 macrophages, which serve as key mediators of both anti-inflammatory and regenerative responses. By secreting cytokines such as IL-10 and TGF-β, M2 macrophages suppress inflammation, while simultaneously releasing growth factors like VEGF and PDGF to stimulate angiogenesis and cellular proliferation—thereby accelerating tissue regeneration [104,105]. In addition, they contribute to extracellular matrix remodeling, facilitate the clearance of necrotic tissue, and promote immune tolerance by shaping a reparative immune microenvironment [106]. Using LPS-stimulated RAW 264.7 cells as an M1-polarized Control, flow cytometry was employed to evaluate macrophage phenotype across treatment groups. Flow cytometric analysis revealed that, following LPS stimulation, the LPS group exhibited 36.43 ± 2.49% CD86^+^ (M1) and 4.55 ± 0.67% CD206^+^ (M2) macrophages (Fig. 6G–I). Compared to BSAMA (29.23 ± 2.45% CD86^+^ and 6.13 ± 0.95% CD206^+^) and HSAMA groups (30.50 ± 1.91% CD86^+^ and 5.01 ± 1.00% CD206^+^), PlasmaMA showed the strongest effect on macrophage polarization, reducing M1 macrophages to 18.03 ± 1.50% and increasing M2 macrophages to 11.03 ± 0.95% (both *p* < 0.0001). To further validate these findings, immunofluorescence staining of CD86 and CD206 was performed in LPS-stimulated RAW 264.7 macrophages. Consistent with the flow cytometry results, PlasmaMA treatment reduced CD86 expression and enhanced CD206 staining compared with BSAMA and HSAMA (Fig. S15). These results suggest that PlasmaMA more effectively modulated macrophage phenotype under inflammatory conditions and may contribute to the establishment of a more pro-reparative immune microenvironment.

Under LPS stimulation, RAW 264.7 macrophages exhibited a significant increase in NO production, reaching 17.78 ± 1.44 μM in the LPS group (Fig. S16E). Treatment with cryogel extracts derived from BSAMA (13.33 ± 0.83 μM, *p* < 0.01), HSAMA (13.62 ± 0.66 μM, *p* < 0.01), and PlasmaMA (9.26 ± 1.00 μM, *p* < 0.001) resulted in a marked reduction in NO release. Notably, PlasmaMA exhibited a significantly stronger inhibitory effect on NO production compared to BSAMA and HSAMA (*p* < 0.01). The concentrations of TNF-α, IL-6, and IL-10 were quantified using ELISA kits (Fig. 6J, K, and S16F). The results showed that under LPS stimulation, the concentrations of TNF-α, IL-6, and IL-10 in the LPS Control group were 302.99 ± 25.42 pg/mL, 322.28 ± 13.03 pg/mL, and 62.96 ± 3.82 pg/mL, respectively. Treatment with the three cryogel extracts resulted in a reduction of pro-inflammatory cytokines TNF-α and IL-6 and an upregulation of the anti-inflammatory cytokine IL-10. Notably, the PlasmaMA group demonstrated the most pronounced immunomodulatory effect, with TNF-α and IL-6 levels reduced to 65.90 ± 8.99 (*p* < 0.0001) and 216.60 ± 6.01 (*p* < 0.001), respectively, while IL-10 levels increased to 216.71 ± 15.53 (*p* < 0.0001). These results underscore the superior anti-inflammatory capacity of PlasmaMA in modulating macrophage responses under inflammatory conditions.

In summary, PlasmaMA cryogels have multifunctional bioactivities that support cell adhesion, proliferation, and migration. In addition, PlasmaMA cryogels support sustained release of endogenous VEGF, promoting robust angiogenesis, and enhancing fibroblast migration. Furthermore, PlasmaMA-derived multicomponent (e.g. albumin, fibronectin) regulate oxidative stress, enhance cellular defense, and exert antioxidant and anti-inflammatory effects, thereby alleviating inflammation and promoting tissue repair.

### 2.4 PlasmaMA cryogels promoted diabetic wound healing and exhibited good biocompatibility *in vivo*

**Fig. 7:**
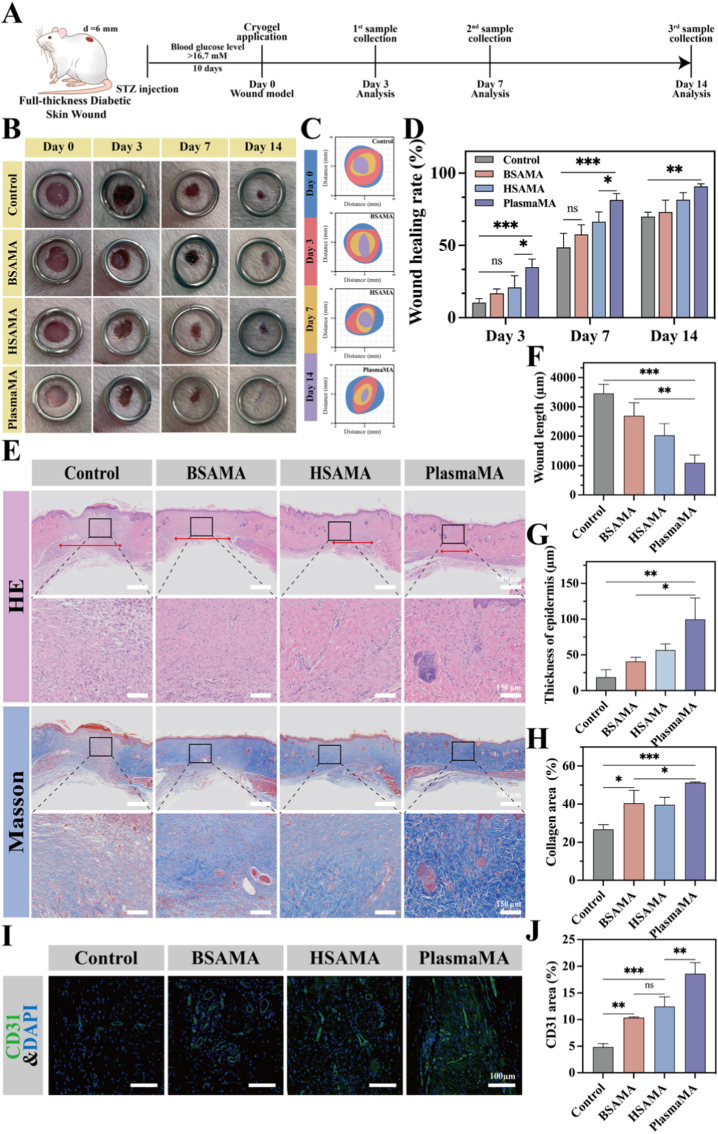
Evaluation of full-thickness wound healing in a diabetic rat skin model following treatment with BSAMA, HSAMA, and PlasmaMA cryogels. (A) Timeline of the animal experiment. (B) Representative images of wounds from the Control, BSAMA, HSAMA, and PlasmaMA groups on days 0, 3, 7, and 14 post-wounding. (C) Wound healing images and (D) quantitative analysis of wound area rate over time. Sections were subjected to histological evaluation. (E) H&E staining and Masson's trichrome staining of wound tissues on day 14, with the red double-headed arrows representing the wound length. (F) Wound length, (G) skin thickness, and (H) quantitative analysis of collagen deposition after 14 days of treatment. Sections were also subjected to (I) immunofluorescence staining (DAPI: blue, CD31: green), scale bar: 100 μm, and (J) quantification of CD31-positive blood vessels per field. ∗*p*< 0.05, ∗∗*p*< 0.01, and ∗∗∗*p* < 0.001 (n = 4, mean ± SD); ns denotes ‘not significant’. (For interpretation of the references to color in this figure legend, the reader is referred to the Web version of this article.)

To evaluate the *in vivo* efficacy of PlasmaMA cryogel in promoting chronic wound healing, a diabetic rat model was established via intraperitoneal injection of streptozotocin (STZ). Rats with non-fasting blood glucose levels exceeding 16.7 mM were considered diabetic. Full-thickness circular wounds (Φ 6 mm) were created on the dorsal region of each rat using a biopsy punch. Cylindrical cryogel samples (Φ 6 × 4 mm^3^) from four groups—Control (PBS), BSAMA, HSAMA, and PlasmaMA—were applied to these wounds, as shown in Fig. 7A. Wound closure was monitored and photographed on days 0, 1, 3, 7, and 14. Throughout the experiment, no signs of infection or adverse complications were observed in any group. As depicted in Fig. 7B–D, the PlasmaMA group exhibited the most effective wound healing rates on days 3, 7 and 14. Notably, the PlasmaMA group achieved a wound closure rate of 90.75 ± 2.98% by day 14, compared to 69.97 ± 3.07% (*p* < 0.01) in the Control group, 73.14 ± 8.21% (*p* < 0.05) in the BSAMA group, and 81.62 ± 4.90% (*p* < 0.05) in the HSAMA group. These results highlight the significant therapeutic potential of PlasmaMA cryogels in accelerating chronic wound repair.

To further evaluate the efficacy of PlasmaMA cryogels in promoting chronic wound healing, histological analysis was conducted. As shown in Fig. 7E, F, and G, H&E staining revealed superior tissue regeneration in the PlasmaMA group, particularly in terms of reduced wound gap and increased epidermal regeneration. The wound length in the PlasmaMA group was 1095.22 ± 267.10 μm, significantly smaller, compared with 3460.28 ± 309.15 μm (*p* < 0.001) in the Control group, 2698.42 ± 441.02 μm (*p* < 0.01) in the BSAMA group, and 2030.05 ± 397.64 μm (*p* < 0.05) in the HSAMA group. Additionally, the PlasmaMA group exhibited the greatest epidermal thickness (99.72 ± 19.98 μm), substantially higher than that observed in the Control (18.66 ± 10.72 μm, *p* < 0.01), BSAMA (40.86 ± 5.71 μm, *p* < 0.05), and HSAMA (56.57 ± 8.80 μm, *p* < 0.05) groups. Throughout the experiment, the inflammatory response remained within a manageable range. These results suggest that PlasmaMA cryogel effectively accelerates wound closure and promotes epithelial regeneration. Additional histological quantification further supported the superior wound-healing performance of PlasmaMA (Fig. S17). Specifically, the PlasmaMA group exhibited the smallest epidermal gap length (731.83 ± 160.21 μm) and the greatest granulation tissue thickness (726.68 ± 65.95 μm), among all groups, further indicating accelerated re-epithelialization and improved wound bed reconstruction, consistent with the pro-regenerative and pro-healing effects observed in the preceding cellular and molecular analyses.

Collagen deposition was evaluated using Masson's trichrome staining. The PlasmaMA group displayed the highest relative collagen content (51.34 ± 3.39%), significantly greater than the Control (26.73 ± 2.45%, *p* < 0.001), BSAMA (40.41 ± 6.78%, *p* < 0.05), and HSAMA (39.64 ± 3.99%, *p* < 0.05) groups, indicating enhanced ECM remodeling (Fig. 7H).

Additionally, angiogenic potential was assessed via immunofluorescence (IF) staining for CD31, a marker of vascular endothelial cells (Fig. 7I). Quantitative analysis (Fig. 7J) revealed that the PlasmaMA group exhibited the highest expression of CD31 (18.63 ± 3.89%), significantly exceeding the Control group (4.84 ± 0.64%, *p* < 0.001), BSAMA group (10.33 ± 1.63%, *p* < 0.01), and HSAMA group (12.46 ± 1.79%, *p* < 0.01). This indicates that PlasmaMA cryogel significantly enhances neovascularization, further supporting its role in facilitating chronic wound healing.

Diabetic wounds, a representative form of chronic wounds, are characterized by a sustained inflammatory milieu throughout the healing process. Consequently, an ideal wound dressing should not only accelerate tissue regeneration but also exert precise control over inflammation. During the inflammatory phase, macrophage polarization dynamically transitions between pro-inflammatory M1 and reparative M2 phenotypes. Excessive M1 activation amplifies inflammatory cascades, while reduced M2 populations further impede tissue repair. On day 3, quantification of inflammatory cells demonstrated a reduction in all three experimental groups compared to the Control group, with the PlasmaMA group exhibiting the most pronounced anti-inflammatory effect (Fig. S18).

**Fig. 8:**
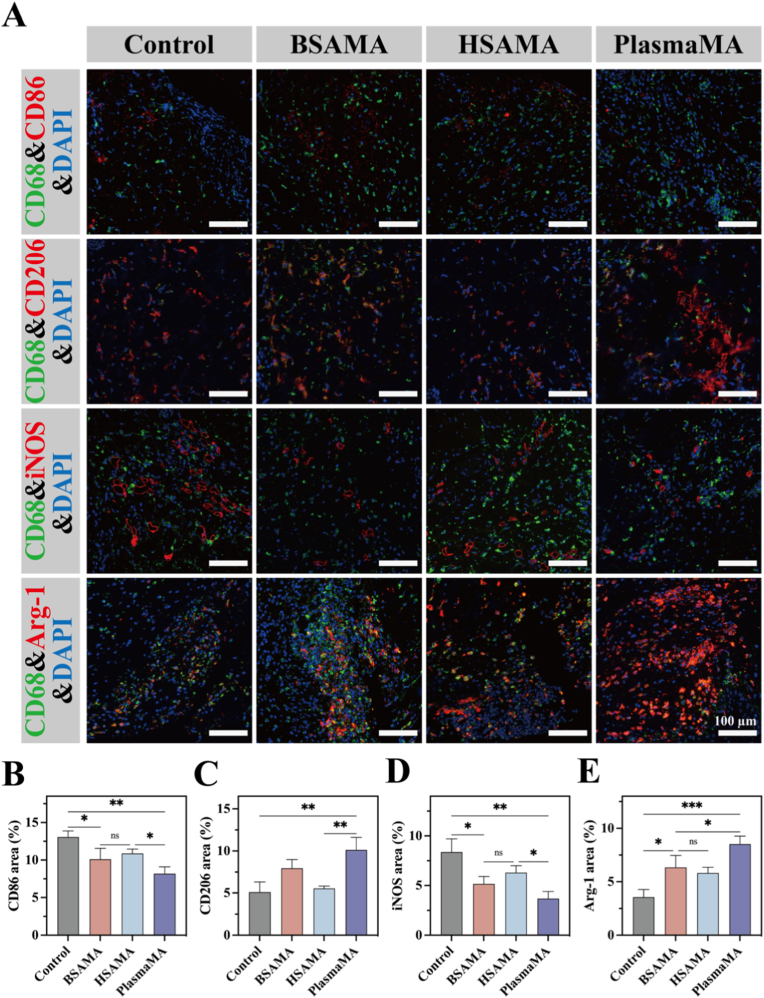
Immunofluorescence staining of diabetic wounds at day 3 post-treatment. (A) Representative immunofluorescence images showing macrophage infiltration (CD68) and polarization markers (CD86, CD206, iNOS, and Arg-1) within wound tissues. Scale bar: 100 μm. (B-E) Quantitative analysis of fluorescence intensity for CD86, CD206, iNOS, and Arg-1 expression. ∗*p*< 0.05, ∗∗*p*< 0.01, and ∗∗∗*p* < 0.001 (n = 4, mean ± SD). ns denotes ‘not significant’.

To assess the inflammation-modulating capacity of the three cryogels in diabetic wound healing, macrophage polarization was analyzed in wound sections harvested on day 3 (Fig. 8A). As shown in Fig. 8B–E, all cryogel-treated groups exhibited a significant reduction in M1 macrophages compared to the Control (CD86^+^ area: 13.07 ± 0.81%). Among them, the PlasmaMA group demonstrated the strongest suppression of M1 polarization, with a CD86^+^ area of 8.19 ± 0.92% (Fig. 8B). Conversely, M2 macrophage populations (CD206^+^) were markedly higher in the PlasmaMA (10.13 ± 1.48%) and BSAMA (7.95 ± 1.04%) groups compared to the HSAMA group (5.53 ± 0.30%) and the Control (5.10 ± 1.04%) (Fig. 8C). This shift in macrophage phenotype from pro-inflammatory M1 to pro-healing M2 was further confirmed by staining for the canonical markers iNOS (M1 marker) and Arg-1 (M2 marker). Treatment with the cryogels resulted in decreased iNOS expression and elevated Arg-1 levels. Importantly, the PlasmaMA group again showed the most pronounced effect, with the lowest iNOS^+^ area (3.66 ± 0.72% vs. 8.37 ± 1.33% in the Control) and the highest Arg-1^+^ area (8.52 ± 0.74% vs. 3.55 ± 0.71% in the Control) (Fig. 8D and E). Collectively, these findings indicate that PlasmaMA cryogel enables favorable inflammatory regulation during the early phase of diabetic wound repair by suppressing M1 activation and promoting M2 polarization. When integrated with the histological findings of accelerated wound closure (HE staining), enhanced collagen deposition (Masson's trichrome), and robust angiogenesis (CD31 staining), PlasmaMA demonstrates a dual capacity for immune modulation and regenerative promotion, underscoring its potential as an advanced wound dressing for chronic wound management.

In subcutaneous implantation models, the host typically recognizes implanted materials as foreign bodies, thereby activating the immune system and triggering a cascade of complex signaling events [107]. In contrast to the wound healing model, which is characterized by a highly inflammatory microenvironment, the subcutaneous implantation model offers a more controlled *in vivo* setting for evaluating the intrinsic degradation behavior of the cryogel. These responses can lead to pronounced inflammation, collagen deposition, and the formation of a fibrotic capsule at the material–tissue interface [108]. Collagen accumulation in the early stage of implantation is widely regarded as a hallmark of foreign body response (FBR) and serves as a critical indicator of the material's compatibility with host tissue [109].

**Fig. 9:**
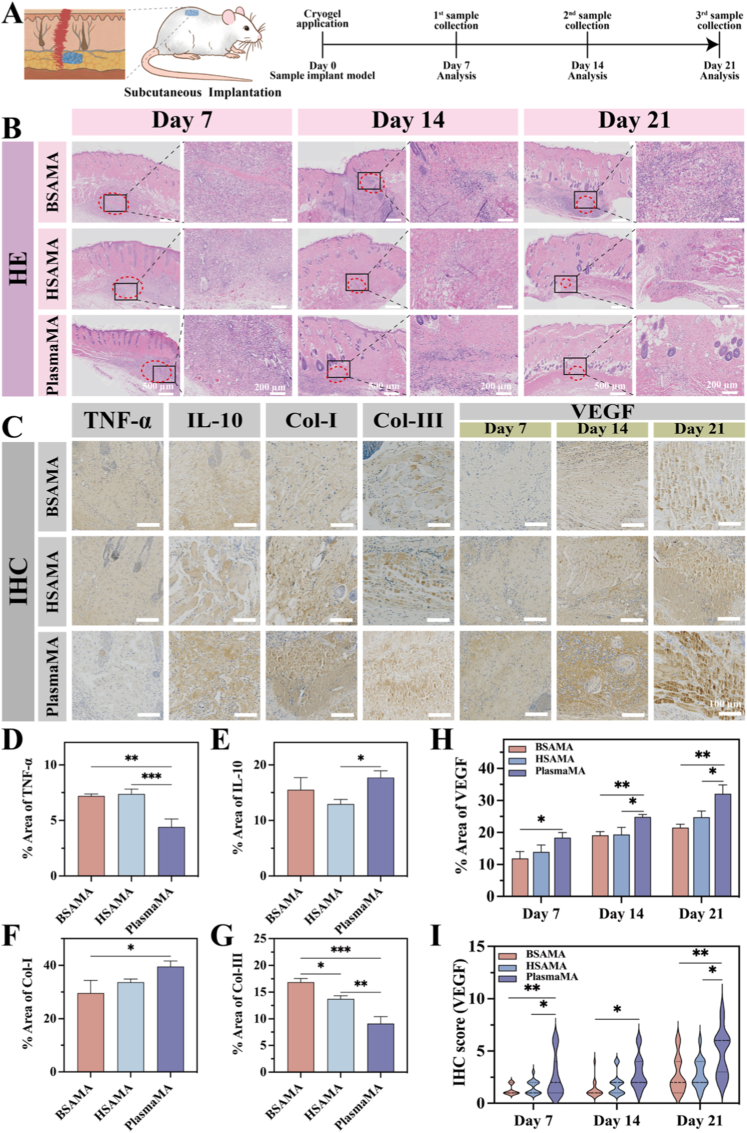
PlasmaMA cryogels facilitate tissue regeneration in a time-dependent manner following subcutaneous implantation. (A) Schematic diagram of the subcutaneous implantation procedure. (B) Histological evaluation of implant sites using H&E staining at 7, 14, and 21 days post-implantation. The red circle marks the approximate contour of the residual cryogel. (C) Immunohistochemical staining for inflammatory and regenerative markers: TNF-α (at day 7), IL-10 (at day 7), Col-I (at day 21), Col-III (at day 21), and VEGF (at 7, 14, and 21 days). Scale bar: 100 μm. (D-H) Quantitative analysis of immunohistochemical staining intensity for TNF-α, IL-10, Col-I, Col-III, and VEGF. (I) VEGF immunohistochemistry (IHC) score combined with immunohistochemical results. ∗*p*< 0.05, ∗∗*p*< 0.01, and ∗∗∗*p* < 0.001 (n = 4, mean ± SD). ns denotes ‘not significant’. (For interpretation of the references to color in this figure legend, the reader is referred to the Web version of this article.)

Based on these considerations, a subcutaneous implantation model was established to systematically assess the biocompatibility and *in vivo* degradation behavior of the PlasmaMA cryogel as a tissue scaffold (Fig. 9A and Fig. S19). Rats were anesthetized with isoflurane, and a 1 cm dorsal incision was made for implantation of cryogel scaffolds. H&E staining (Fig. 9B) revealed distinct host responses, degradation kinetics, and tissue integration among the three cryogels. The BSAMA group exhibited relatively slow degradation and persistent inflammatory encapsulation throughout the observation period, indicating limited tissue remodeling. The HSAMA group triggered a more pronounced early inflammatory response, which promoted progressive material degradation and cellular infiltration in the later stages. PlasmaMA exhibited the most favorable overall profile, likely attributable to the intrinsic compositional complexity of plasma-derived proteins, which furnish both structural support and multifaceted biological cues for host–material interactions. The moderate inflammatory response observed at day 7 may establish a permissive microenvironment that facilitates subsequent cell recruitment and angiogenesis, whereas the progressive degradation at later stages enables effective tissue ingrowth and gradual scaffold replacement.

Successful skin wound healing relies heavily on the formation of new tissue. As the primary component of the extracellular matrix, collagen plays a pivotal role in neovascularization and epithelial regeneration [99]. Masson's trichrome staining and subsequent quantitative analysis of collagen deposition (Fig. S20) revealed a time-dependent increase in collagen accumulation across all groups. By day 7 post-implantation, collagen deposition was comparable among the three groups—approximately 30%—falling within the typical range observed during early tissue repair. However, by day 14, the PlasmaMA group exhibited significantly greater collagen deposition (57.25 ± 6.55%) compared to BSAMA (44.98 ± 3.70%, *p* < 0.01). This trend persisted through day 21, with the PlasmaMA group reaching 64.13 ± 4.96%, significantly exceeding the BSAMA group (55.19 ± 1.89%, *p* < 0.05), thereby indicating sustained enhancement in matrix remodeling.

Histological evaluation revealed distinct patterns of tissue integration. In the BSAMA group, collagen was primarily confined to a fibrous capsule surrounding the implant, with limited intramaterial infiltration, suggesting poor tissue integration. The HSAMA group showed a more intense early inflammatory response, followed by progressive replacement with collagenous tissue, indicative of moderate repair activity. In contrast, the PlasmaMA group demonstrated superior outcomes: abundant, dense, and well-organized collagen matrix formation, along with extensive host cell infiltration and constructive remodeling. Although early inflammatory infiltration was observed in all groups, the PlasmaMA group displayed a relatively mild immune response, and the scaffold was effectively replaced by newly formed connective tissue, resulting in superior tissue integration and minimal scarring. Accordingly, timely collagen deposition and its spatial organization are critical metrics for evaluating wound healing quality. Within this context, PlasmaMA cryogels fostered a regenerative microenvironment conducive to constructive remodeling—likely facilitated by their intrinsic antioxidant properties. The prominent collagen accumulation and neovascular formation observed in the Masson-stained sections further underscore their strong potential for enhancing tissue regeneration.

Although differences were observed between the *in vitro* and *in vivo* degradation behaviors of the cryogels, such discrepancies are commonly reported for biomaterial scaffolds evaluated under distinct biological environments. *In vitro* degradation under 3D cell culture conditions is primarily governed by hydrolytic processes and limited cell-material interactions, whereas subcutaneous implantation *in vivo* involves the coordinated participation of immune cells, enzymatic activity, and dynamic tissue remodeling. These factors collectively modulate scaffold degradation kinetics in a physiological context. Notably, the *in vivo* degradation profile indicates that the cryogels can appropriately respond to biological cues, allowing for their gradual resorption and progressive replacement by newly formed tissue.

Immunohistochemical (IHC) analysis (Fig. 9C) confirmed the absence of severe inflammatory reactions across all groups, with no pathological abnormalities detected in major organs (Fig. S21), affirming excellent systemic biocompatibility. Quantitative analysis of inflammatory cytokines TNF-α and IL-10 (Fig. 9D and E) demonstrated that the PlasmaMA group exhibited significantly lower TNF-α expression (4.41 ± 2.71%) compared to BSAMA (7.19 ± 2.18%, *p* < 0.01) and HSAMA (7.38 ± 2.44%, *p* < 0.001). Conversely, IL-10 expression was elevated in PlasmaMA (17.72 ± 2.23%), exceeding levels in BSAMA (15.52 ± 3.21%) and HSAMA (12.95 ± 1.82%, *p* < 0.05). This anti-inflammatory (reduced TNF-α) and pro-regenerative (elevated IL-10) cytokine profile underscores the superior immunomodulatory capacity of PlasmaMA.

Collagen maturation was assessed via immunostaining for type I (Col-I) and type III (Col-III) collagen (Fig. 9F and G). During the remodeling phase of wound healing, collagen composition transitions from Col-III-rich granulation tissue to mature Col-I-dominant tissue. By day 21, the PlasmaMA group exhibited a significantly higher Col-I content (39.57 ± 2.07%) than the BSAMA group (29.61 ± 4.74%, *p* < 0.05), accompanied by a markedly lower Col-III content (9.11 ± 1.31%) compared with both BSAMA (16.86 ± 0.68%, *p* < 0.001) and HSAMA (13.75 ± 0.57%, *p* < 0.01) groups. The Col-I/Col-III ratio, a critical indicator of healing quality and extracellular matrix remodeling, was 1.76 ± 0.28 for BSAMA, 2.45 ± 0.09 for HSAMA, and 4.35 ± 0.23 for PlasmaMA—the highest among all groups—indicating advanced collagen maturation and structural integration (Fig. S22). These results support more advanced collagen maturation and extracellular matrix remodeling in the PlasmaMA implantation group [110].

VEGF expression, a critical marker for neovascularization, was also evaluated to assess the effect of the materials on angiogenesis. Quantitative analysis and IHC scoring (Fig. 9H and I) revealed that the PlasmaMA group exhibited the highest VEGF expression on days 7, 14 and 21. Especially, the angiogenic capacity of PlasmaMA (32.09 ± 4.76%) was significantly higher than that of the BSAMA group (21.49 ± 3.08%, *p* < 0.01) and the HSAMA group (24.77 ± 3.96%, *p* < 0.05) at day 21, confirming the superior angiogenic capacity of PlasmaMA. Abundant, well-organized collagen deposition, together with a high Col-I/Col-III ratio and elevated VEGF expression could be indicative of regenerative repair rather than fibrotic scarring. In parallel, the gradual, time-dependent degradation of PlasmaMA maintained early structural support while permitting progressive replacement by nascent tissue, ensuring durable regenerative outcomes.

PlasmaMA cryogel significantly promotes angiogenesis, cell migration, and collagen deposition, likely attributed to its enrichment with bioactive proteins and antioxidant components. These properties, combined with its excellent biocompatibility, favorable immunomodulatory profile, and controlled degradability, position PlasmaMA as a promising scaffold for tissue engineering. In both *in vitro* and *in vivo* models, PlasmaMA cryogel demonstrates exceptional performance in chronic wound healing and tissue regeneration, maintaining structural integrity while promoting functional integration. Collectively, these findings underscore its potential as a next-generation biomaterial for regenerative applications. To substantiate these findings, larger-scale animal experiments are still essential to ensure reproducibility. Rigorous long-term evaluations will be required to define its safety and efficacy across diabetic and other chronic disease models. Future investigations should focus on evaluating the feasibility of PlasmaMA as a customizable scaffold while establishing scalable manufacturing strategies to support clinical translation. Rigorous long-term evaluations will further define its therapeutic utility and address critical questions regarding durability, immune compatibility, and integration in complex pathological environments.

## 3 Conclusion

This study introduces a novel human plasma-derived cryogel, PlasmaMA, as a multifunctional scaffold for accelerating chronic wound healing and tissue regeneration. By leveraging the intrinsic bioactive components of human plasma, PlasmaMA integrates structural, biochemical, and immunomodulatory properties to create a dynamic microenvironment conducive to tissue repair. The scaffold's highly interconnected macroporous architecture, combined with its capacity to retain endogenous growth factors and ECM proteins (e.g., fibronectin, fibrinogen), synergistically enhances cell adhesion, angiogenesis, and collagen maturation. *In vitro* evaluations demonstrate its superior antioxidant and anti-inflammatory capabilities, effectively mitigating oxidative stress and suppressing pro-inflammatory macrophage polarization. *In vivo* studies further validate PlasmaMA's efficacy in diabetic wound healing, with accelerated wound closure, robust neovascularization, and enhanced extracellular matrix remodeling compared to conventional albumin-based scaffolds. Mechanistically, the scaffold's design bridges static biomaterials with adaptive, patient-tailored therapies by mimicking native ECM functionality and delivering bioactive signals in a spatiotemporally controlled manner. As a customized platform, PlasmaMA addresses critical unmet needs in regenerative medicine, offering a transformative strategy for chronic wound management and precision tissue engineering. Future studies will focus on optimizing scalability and exploring its broader applications in diverse pathological conditions.

## 4 Experiments and methods

### 4.1 Materials

Human umbilical vein endothelial cells (HUVECs, RRID: CVCL_2959) were obtained from the American Type Culture Collection (ATCC, Cat. No. PCS-100-013). NCTC clone 929 (L929, RRID: CVCL_4237) fibroblasts and mouse monocyte-macrophage-like cells (RAW 264.7, RRID: CVCL_0493) were purchased from Wuhan Pricella Biotechnology Co., Ltd. (Wuhan, China) (L929s: Cat. No. CL-0137; RAW 264.7: Cat. No. CL-0190). Human plasma was sourced from Kejing Biological Technology Co., Ltd. (Jiangsu, China). Bovine serum albumin (BSA), human serum albumin (HSA), methacrylic anhydride (MAA, 94%), 3-(trimethylsilyl)propionic-2,2,3,3-d_4_ acid sodium salt (TMSP), and 2,4,6-trinitrobenzenesulfonic acid (TNBS) solution were procured from Sigma-Aldrich (Shanghai, China). Ammonium persulfate (APS, >98%) and N,N,N′,N'-tetramethylethylenediamine (TEMED, ≥99.5%) were obtained from Rhawn, Shanghai Yi En Chemical Technology Co., Ltd. (Shanghai, China). Dulbecco's Modified Eagle Medium (DMEM), fetal bovine serum (FBS), penicillin/streptomycin (P/S), Trypsin-EDTA, Rhodamine phalloidin, Calcein-AM, and ethidium homodimer-1 (EthD-1) were supplied by Thermo Fisher Scientific Inc. (Waltham, MA, USA). Proteinase K, streptozotocin (STZ), and Triton X-100 were purchased from Shanghai Aladdin Biochemical Technology Co., Ltd. (Shanghai, China). The Cell Counting Kit-8 (CCK-8) was acquired from Dojindo Molecular Technologies (Kumamoto, Japan).

### 4.2 Human plasma methacryloyl cryogel preparation

#### 4.2.1 Synthesis of human plasma methacryloyl and ^1^H-NMR analysis

BSAMA and HSAMA were synthesized according to protocols described in our previous studies (BSA: A1933, Sigma; HSA: A1653, Sigma) [7,8]. In contrast, the synthesis of human PlasmaMA followed a modified procedure. In brief, human plasma (100 mL) was first thawed overnight at 4 °C and then gently stirred at room temperature. The pH of the solution was adjusted to approximately 8.0 prior to the gradual addition of methacrylic anhydride (MAA) at a molar ratio of around 2.2:1 (MAA to reactive groups). The reaction was maintained at a pH of 7.4–8.0 throughout and allowed to proceed for 3–4 h until complete consumption of MAA. The resulting mixture was filtered and subsequently purified by dialysis against deionized water at 37 °C for 6–8 h using a MasterFlex® tangential flow filtration (TFF) system equipped with a Pellicon® 2 cassette (Darmstadt, Germany) and a 10 kDa Biomax membrane. The purified product was subsequently lyophilized to obtain the final PlasmaMA. This purification step was used primarily to remove unreacted small molecules and by-products while favoring the retention of plasma-derived macromolecular components.

The successful methacrylation of BSAMA, HSAMA, and PlasmaMA was confirmed by proton nuclear magnetic resonance (^1^H-NMR) spectroscopy. Each sample (around 8 mg) of BSA, BSAMA, HSA, HSAMA, Plasma, and PlasmaMA was dissolved in deuterium oxide (D_2_O) containing 3-(trimethylsilyl)propionic-2,2,3,3-d_4_ acid sodium salt (TMSP, 0.18 mg/mL; 935638, Sigma) as an internal standard. Spectra were acquired using a Bruker Avance-I 400 MHz spectrometer (QUANTUM-I-400MHz, Weinheim, Germany). The degree of methacryloyl functionalization was quantified by integrating the characteristic methacryloyl peak (^1^H at around 5.5 ppm) relative to the signal (9H) at 0 ppm with TMSP serving as the reference standard.

#### 4.2.2 Fourier transform infrared (FT-IR) spectra

Fourier-transform infrared (FT-IR) spectroscopy was employed to characterize the functional groups of BSA, BSAMA, HSA, HSAMA, Plasma, and PlasmaMA. Prior to analysis, all samples were lyophilized for 24 h using a low-temperature freeze dryer. FT-IR spectra were collected using a Bruker Tensor II spectrometer (Tensor II, Ettlingen, Germany) over the spectral range of 4000–1000 cm^−1^.

#### 4.2.3 Determination of the methacryloyl functionalization degree of BSAMA, HSAMA and PlasmaMA

The degree of free amino group methacryloyl functionalization in BSAMA, HSAMA, and PlasmaMA was quantified using the 2,4,6-trinitrobenzenesulfonic acid (TNBS; P2297, Sigma) assay. A glycine standard curve was first established by dissolving glycine in 0.1 M sodium bicarbonate buffer at a concentration of 0, 5, 10, 50, 100, 150, and 200 μg/mL. Concurrently, BSA, BSAMA, HSA, HSAMA, Plasma, and PlasmaMA were each dissolved in 0.1 M sodium bicarbonate buffer at a final concentration of 1.6 mg/mL. For each sample, 0.25 mL of a 0.1% (w/w) TNBS solution was added to 0.25 mL of each protein solution and incubated at 37 °C for 2 h. The reaction was terminated by the addition of 0.25 mL of 1 M HCl and 0.25 mL of 10% (w/w) SDS, followed by vigorous vortexing. Absorbance was measured at 335 nm, and the concentration of residual free amino groups was calculated based on the glycine calibration curve.

To quantify the extent of hydroxyl group modification in BSAMA, HSAMA, and PlasmaMA, a Fe(III)-hydroxamic acid-based assay was employed. BSA, BSAMA, HSA, HSAMA, Plasma, and PlasmaMA were dissolved separately in DPBS (pH 7.2) at a concentration of 50 mg/mL. Hydroxylamine hydrochloride (HAHC, 0.5 M; HX0770, Sigma) and Fe(III) perchlorate (0.5 M; T283498, Aladdin) in 0.5 M HCl were freshly prepared. Each sample (200 μL) was mixed with 100 μL of HAHC and 100 μL of 1 M NaOH and then incubated at 37 °C for 10 min. Subsequently, 550 μL of 0.5 M HCl and 50 μL of a Fe(III) solution were added, thoroughly mixed, and centrifuged at 6000 rpm for 5 min at room temperature. A 200 μL aliquot of the supernatant was transferred to a 96-well plate, and absorbance was measured at 500 nm. To establish a standard curve, acetohydroxamic acid (AHA; 159034, Sigma) was prepared at concentrations of 2.5 × 10^−4^, 5 × 10^−4^, 6.25 × 10^−4^, 1.25 × 10^−3^, 2.5 × 10^−3^, and 5 × 10^−3^ mol/L in deionized water. Equal volumes of AHA standard and Fe(III) solutions were mixed and treated identically. The resulting absorbance values were used to generate a calibration curve, from which the amount of methacrylated groups in each sample was determined.

#### 4.2.4 Far-UV CD spectroscopy

To investigate the effects of methacrylation on protein secondary structure, far-UV circular dichroism (CD, Chirascan Plus, Leatherhead, UK) spectroscopy was performed. BSA, BSAMA, HSA, HSAMA, Plasma, and PlasmaMA were each dissolved in deionized water at a concentration of 0.05 mg/mL. UV spectral scans were conducted over the wavelength range of 180–260 nm. To ensure structural stability and minimize degradation, all sample solutions were freshly prepared 4 h prior to measurement. During analysis, 200 μL of each solution was transferred into a 1 mm pathlength quartz cuvette, which was pre-rinsed twice with the corresponding sample solution to eliminate contamination and ensure accuracy [8].

#### 4.2.5 SDS-PAGE

Sodium dodecyl sulfate–polyacrylamide gel electrophoresis (SDS-PAGE) was performed to analyze the molecular weight profiles of BSA, BSAMA, HSA, HSAMA, Plasma, and PlasmaMA. Each sample was dissolved in PBS at a concentration of 1 mg/mL. Solutions were mixed with 5 × SDS-PAGE loading buffer at a volume ratio of 4:1 and denatured by heating at 100 °C for 5 min. Following centrifugation at 8000×*g* for 5 min, 10 μL of the supernatant was loaded into the wells of the SDS-PAGE gel. Electrophoresis was conducted at 80 V through the stacking gel and 120 V through the resolving gel. After separation, proteins were visualized using Coomassie Brilliant Blue staining.

#### 4.2.6 Proteomics analysis

Proteomic analysis was conducted by BestMS (Qingdao BestMS Biotechnology Co., Ltd., Qingdao, China) using their standard proteomics platform.

#### 4.2.7 Preparation of BSAMA, HSAMA and PlasmaMA cryogels

To fabricate 3% cryogel precursor solutions, 30 mg of BSAMA, HSAMA, or PlasmaMA was dissolved in 945 μL of PBS. TEMED (0.1% w/v) and APS (0.5% w/v) were used as the catalyst and initiator, respectively. The precursor solutions were mixed with TEMED and APS in an ice bath, then immediately transferred into pre-cooled silicone molds and incubated at −20 °C for 48 h to yield stable cylindrical cryogels (diameter: 6 mm; height: 4 mm) with varying compositions.

The resulting cryogels were either thawed at room temperature for immediate use or stored at −20 °C for long-term preservation. For sterile cryogel preparation, precursor solutions were filtered through a 0.22 μm sterile syringe filter (331011, Nest) under a biosafety cabinet. Sterile polymerization was achieved by adding TEMED and APS under aseptic conditions, following the same formulation. The resulting sterile cryogels were stored in sterile containers to prevent microbial contamination.

### 4.3 Characterization of BSAMA, HSAMA, and PlasmaMA cryogels

#### 4.3.1 Morphology observation

Scanning Electron Microscopy (SEM): The microstructural morphology of the cryogel scaffolds was examined using a field-emission scanning electron microscope (SU8010, Hitachi, Japan) at an accelerating voltage of 5 kV. Prior to imaging, samples were freeze-dried at −80 °C and sputter-coated with a thin layer of platinum to enhance conductivity [111].

Confocal Laser Scanning Microscopy (CLSM): Freeze-dried cryogels were rehydrated in PBS (pH 7.2) until equilibrium swelling was reached. Excess surface moisture was gently removed using lint-free tissue. The samples were then stained with DAPI for 15 min and subsequently rinsed with PBS to eliminate unbound dye. Pore architecture and internal microstructures were visualized using a confocal laser scanning microscope (A1, Nikon, Japan) [112].

#### 4.3.2 Pore interconnectivity and swelling ratio

The cryogel samples were immersed in PBS for 10 min to reach equilibrium swelling, after which the initial weight was recorded (M_1_). After the loose water in samples were completely removed with Kimwipes, samples were weighed again (M_2_). The pore interconnectivity (PI) was subsequently calculated using the following Eq. (1):

(1) $$\text{Pore}\;\text{interconnectivity}\;\left(\;\text{PI},\%\right)=\frac{M_{1}-{\;M}_{2}}{M_{1}}\times 100\%$$

For swelling ratio analysis, cryogels were freeze-dried for 24 h and weighed (M_0_). Subsequently, the dried cryogels were immersed in PBS, and their weights (M_t_) were recorded at predetermined time points (0, 15, 30, 45, 60, 90, 120, 150, 180, and 240 min) until equilibrium swelling was reached. The swelling ratio was calculated using the following Eq. (2):

(2) $$\text{Swelling}\;\text{ratio}\;\left(\;\text{SR},\%\right)=\frac{M_{t}-{\;M}_{0}}{M_{0}}\times 100\%$$

#### 4.3.3 Degradation assay

To evaluate the *in vitro* stability of BSAMA, HSAMA, and PlasmaMA cryogels, their degradation behavior in PBS was assessed. Individual cryogel samples were placed in centrifuge tubes containing 1 mL of PBS (pH 7.2) and incubated at 37 °C with continuous shaking at 150 rpm. The samples were weighed at predetermined time points (days 0, 1, 2, 3, 7, 10, and 14) to monitor their degradation over time.

Enzymatic degradation was investigated by incubating the cryogels in enzyme solutions. Each cryogel was first immersed in PBS for 10 min to allow equilibrium swelling. Excess surface moisture was gently removed using a lint-free tissue, and the sample was weighed (W_0_). The cryogels were then placed in 1 mL of a Proteinase K solution (0.01 mg/mL) or a Trypsin solution (0.1%) and incubated at 37 °C with shaking at 150 rpm. At designated time intervals, the samples were removed, and their weights (W_t_) were recorded. The degradation rate was calculated by determining the residual mass percentage using the following Eq. (3):

(3) $$\text{Residual}\;\text{ratio}\;\left(\;\text{RR},\%\right)=\frac{W_{t}}{W_{0}}\times 100\%$$

To evaluate the potential of cryogels as bioscaffolds, L929s (2 × 10^5^ cells/cryogel) and HUVECs (1.5 × 10^5^ cells/cryogel) were seeded separately onto the surface of sterile BSAMA, HSAMA, and PlasmaMA cryogels [53]. The samples were weighed at predetermined time points (days 0, 7, 14, 21, 28, 35, and 42) to monitor their residual mass percentage over time.

#### 4.3.4 Mechanical property

Cyclic compression tests were performed using a universal testing machine (Instron, 5944, Massachusetts, USA) to evaluate the mechanical properties of the cryogels. BSAMA, HSAMA, and PlasmaMA cryogels (Φ 6 × 4 mm^3^) were fully swollen in PBS prior to testing and subjected to 10 compression cycles at 80% strain. Compressive stress-strain curves were generated at a compression rate of 10 mm min^−1^ using a Universal Testing Machine.

#### 4.3.5 Rheological analysis

The rheological properties of BSAMA, HSAMA, and PlasmaMA cryogels at equilibrium swelling were assessed at room temperature using a TA DHR-2 rheometer (New Castle, Delaware, USA). BSAMA, HSAMA, and PlasmaMA cryogels (Φ 8 × 5 mm^3^) were pre-swollen in PBS to equilibrium. A strain amplitude sweep was first performed at a constant frequency of 1 Hz over a strain range of 0.1% to 10% to identify the linear viscoelastic region. Subsequently, a frequency sweep was conducted at a fixed strain of 0.2%, with the frequency varied from 0.1 to 10 Hz.

### 4.4 *In vitro* biocompatibility

#### 4.4.1 Cell seeding

To evaluate the biocompatibility of BSAMA, HSAMA, and PlasmaMA cryogels, HUVECs and L929s were cultured on sterile cryogels. Each cryogel was placed in an individual well of a sterile culture plate, pre-swollen in PBS, and rinsed with PBS five times. Excess surface water was gently removed by compression. Subsequently, the cryogels were then equilibrated in Dulbecco's Modified Eagle Medium (DMEM; 11965092, Gibco) supplemented with 10% fetal bovine serum (FBS; A3160901, Gibco) and 1% penicillin–streptomycin (P/S; 15240062, Gibco). Following removal of excess medium, a defined volume of cell suspension was gently deposited onto each cryogel. The constructs were incubated at 37 °C in a humidified atmosphere containing 5% CO_2_ for 4 h to allow cell attachment, after which additional culture medium was added. The culture medium was replaced every other day throughout the experiment. All cultures were incubated at 37 °C in a humidified atmosphere of 5% CO2, with medium changes performed regularly to sustain cell viability.

HUVECs (RRID: CVCL_2959) were obtained from ATCC (Manassas, USA; Cat. No. PCS-100-013) in February 2024. L929s (RRID: CVCL_4237) and RAW264.7 macrophages (RRID: CVCL_0493) were purchased from Procell Life Science & Technology Co., Ltd. (Wuhan, China; Cat. No. CL-0137 and CL-0190, respectively) in April 2024 and August 2024. All cell lines were confirmed to be free from mycoplasma contamination prior to use. Subsequent experiments were carried out when cells reached logarithmic growth phase (approximately 90% confluence). All procedures were conducted under sterile conditions to ensure experimental reproducibility and cellular integrity.

#### 4.4.2 Cell adhesion assay

To assess cell adhesion, L929s (1 × 10^5^ cells/cryogel) and HUVECs (1 × 10^5^ cells/cryogel) were separately seeded onto sterile BSAMA, HSAMA, and PlasmaMA cryogels. Cells cultured in 12-well plates served as the two-dimensional (2D) Control. After incubation at 37 °C with 5% CO_2_ for 2.5 h, non-adherent cells and residual moisture of cryogels were gently rinsed away with PBS. Cell viability was subsequently assessed using the Cell Counting Kit-8 (CCK-8) assay.

To further evaluate the adhesive properties of the three cryogels, 1 × 10^6^ L929s and HUVECs were independently seeded onto each cryogel and incubated for 4 h at 37 °C. After 4 h of incubation, non-adherent cells were gently removed by PBS washing. The adherent cells on the surfaces of the cryogels were stained with 2 μM Calcein-AM (C3099, Invitrogen) for 30 min at 37 °C in the dark to assess viability. Samples were then washed once with PBS and fixed with 4% paraformaldehyde for 15 min at room temperature. Following fixation, samples were washed with PBS, permeabilized using 0.1% Triton X-100 for 5 min, washed again, and blocked with 1% BSA for 30 min. Rhodamine–Phalloidin (1:400 dilution in PBS; R415, Invitrogen) was applied and incubated for 1 h in the dark to stain F-actin. All staining procedures were performed with the cryogels. Finally, cells were detached using cell scrapers, washed with PBS, and analyzed by flow cytometry to quantify both the number of viable adherent cells and the extent of cytoskeletal remodeling.

To investigate whether the pro-adhesive effect of PlasmaMA cryogels is mediated by integrin β1, a blocking assay was conducted. HUVECs (1 × 10^6^ cells/cryogel) were seeded onto 3D PlasmaMA cryogels and divided into two groups: (i) untreated Control and (ii) pretreated with 2 μg/mL CD29 (integrin β1) blocking antibody (A23497, ABclonal). After 4 h of incubation, non-adherent cells were gently removed by PBS washing. The remaining adherent cells were stained with Calcein-AM for 30 min at 37 °C, followed by PBS washing. Cells were then enzymatically detached from the cryogels using Trypsin-EDTA, washed twice with PBS, and subjected to flow cytometry to quantify fluorescence intensity.

#### 4.4.3 Live/dead assay

L929s (1 × 10^4^ cells/cryogel) were cultured on sterile cryogels to assess cell compatibility over time. Live/dead staining was conducted at designated time points (Day 1, 3, and 5). Each cryogel was placed into a separate well of a sterile culture plate. Viable cells were visualized using 2 μM Calcein-AM, while dead cells were identified using 4 μM EthD-1. Samples were incubated at 37 °C with 5% CO_2_ for 45 min, followed by CLSM imaging to assess cell viability.

#### 4.4.4 Cell viability assay

The proliferation of L929s and HUVECs on cryogels was evaluated using the CCK-8 assay. L929s (5 × 10^4^ cells/cryogel) and HUVECs (4 × 10^4^ cells/cryogel) were separately seeded onto sterile BSAMA, HSAMA, and PlasmaMA cryogels and co-cultured for 1, 3, and 5 days. At each time point, the culture medium was carefully removed, and the cryogels were gently washed with PBS to eliminate non-adherent cells. Subsequently, 350 μL of complete medium supplemented with 10% CCK-8 reagent was added to each well, and the samples were incubated for 90 min at 37 °C. After incubation, 100 μL of the supernatant was transferred to a 96-well plate, and the absorbance was measured at 450 nm using a microplate reader (EP0CH2, BioTek).

#### 4.4.5 Cell morphology

L929s (1.5 × 10^4^ cells/cryogel) and HUVECs (1 × 10^4^ cells/cryogel) were separately seeded onto sterile BSAMA, HSAMA, and PlasmaMA cryogels and co-cultured for 5 days. Following incubation, the cell constructs were gently rinsed with PBS, and 400 μL of 4% paraformaldehyde was added for fixation at room temperature for 15 min. After fixation, samples were washed with PBS for 5 min and subsequently permeabilized with 0.1% Triton X-100 for 5 min. Following a final PBS rinse, Rhodamine–Phalloidin was applied to stain the actin cytoskeleton for 1 h in the dark. Cell nuclei were then counterstained with DAPI (C1005, Beyotime) for 5 min. Cell morphology and attachment were visualized using CLSM.

#### 4.4.6 Hemolysis ratio

Whole blood was collected from the ear vein of healthy rats and anticoagulated with sodium citrate (EDTAK_2_; Hebei Kangweishi Medical Technology Co., Ltd., Hebei, China). The blood was centrifuged at 1000 rpm for 15 min to isolate red blood cells by removing the platelet-rich plasma and white blood cell layers. The harvested red blood cells were washed with PBS and centrifuged at 2000 rpm for 5 min; the supernatant was discarded. The red blood cells were then resuspended in PBS to obtain a 2% RBC suspension. Swollen cryogel samples were placed into 1.5 mL centrifuge tubes, followed by the addition of 0.5 mL of the 2% RBC suspension and 0.5 mL of PBS. The mixtures were incubated at room temperature for 1 h. For the Control groups, 0.5 mL of the 2% RBC suspension was mixed with either 0.5 mL of DI water (positive Control) or 0.5 mL of PBS (negative Control). After incubation, all samples were centrifuged at 3000 rpm for 5 min. A 100 μL aliquot of each supernatant was transferred into a 96-well plate, and absorbance was measured at 540 nm using a microplate reader [113]. The hemolysis rate was calculated using the following Eq. (4):

(4) $$\text{Hemolysis}\;\text{ratio}\;\left(\;\text{HR},\%\right)=\frac{{OD}_{Sample}-{OD}_{Negative}}{{OD}_{Positive}-{OD}_{Negative}}\times 100\%$$

#### 4.4.7 VEGF cumulative release

VEGF release from BSAMA, HSAMA, and PlasmaMA cryogels was quantified using an ELISA kit (EH0327, FineTest). Each cryogel was immersed in a centrifuge tube containing 1 mL of PBS (pH 7.2) and incubated at 37 °C with shaking at 150 rpm. At predetermined time points (days 3, 7, and 14), 150 μL of each extract was collected and stored at −20 °C for analysis. An equal volume of fresh PBS (150 μL) was then replenished to maintain a constant volume. After all time points were completed, VEGF concentrations were measured according to the manufacturer's instructions using the ELISA kit.

#### 4.4.8 Tube formation assay

Each cryogel was placed in a centrifuge tube containing 1 mL of DMEM and incubated at 37 °C with shaking at 150 rpm for 3 days. The resulting extract was filtered through a 0.22 μm syringe filter. Matrigel (C0372, Beyotime) was thawed one day in advance and coated onto a pre-cooled 24-well plate (20 μL per well), evenly spread, and stored at 4 °C overnight. On the following day, the solidified Matrigel was transferred to a cell culture incubator to stabilize its structure. HUVECs (1.5 × 10^5^ cells/well) were seeded onto the Matrigel surface. The culture medium was prepared by mixing each cryogel extract with complete endothelial growth medium at a 1:1 ratio. Tube formation was observed at 5- and 10-h post-seeding using an inverted biological microscope (BDS400, Optec Technology), and representative images were captured for analysis.

#### 4.4.9 Wound healing assay

L929s (4 × 10^4^ cells/well) were seeded in a 24-well plate and cultured until they formed a confluent monolayer. A scratch was introduced using a 200 μL pipette tip to create a wound in the monolayer. The scratched monolayers were then co-cultured with sterile BSAMA, HSAMA, and PlasmaMA cryogels in DMEM medium using a Transwell system (725321, Nest). After 24 h of incubation, cell migration was observed, and representative images were captured using an inverted biological microscope.

#### 4.4.10 qPCR assay

The gene expression profiles of L929s and HUVECs after co-culture with sterile BSAMA, HSAMA, and PlasmaMA cryogels were analyzed using quantitative real-time PCR (qPCR). L929s (7 × 10^5^ cells/cryogel) and HUVECs (5 × 10^5^ cells/cryogel) were separately seeded on sterile BSAMA, HSAMA, and PlasmaMA cryogels in 48-well plates. After 4 days of co-culture, total RNA was extracted using the RNA Rapid Purification Kit (RN001, ES Science). The RNA was then reverse transcribed into complementary DNA (cDNA) using the Prime Script™ RT Reagent Kit (RR037A, Takara). qPCR was performed using the corresponding primers and TB Green® Premix Ex Taq™ II (RR820Q, Takara) on a LightCycler® 480 system. The primer sequences are provided in Table S3.

#### 4.4.11 *In vitro* antioxidant activity

Antioxidant Capacity Evaluation: The antioxidant capacities of BSAMA, HSAMA, and PlasmaMA cryogels were assessed using the 2,2′-azino-bis (3-ethylbenzothiazoline-6-sulfonic acid) (ABTS) and 2,2-Diphenyl-1-picrylhydrazyl (DPPH) free radical scavenging assays. Dried cryogel samples were immersed in the respective analytical reagent for 2 h to allow interaction. Subsequently, 100 μL of each sample was transferred to a 96-well plate. Absorbance was measured at 414 nm for the ABTS assay and at 516 nm for the DPPH assay to assess radical scavenging activity.

Cell Protection Assay: The protective effects of BSAMA, HSAMA, and PlasmaMA cryogels on cell survival under oxidative stress were evaluated by assessing cell viability following treatment with 1 mM H_2_O_2_. To prepare each cryogel extract, each cryogel type was immersed in 1 mL of a 100 mM H_2_O_2_ solution for 3 days. L929s (1 × 10^4^ cells/well) and HUVECs (1 × 10^4^ cells/well) were separately seeded in 96-well plates and allowed to adhere overnight. On the following day, the cells were treated with the cryogel extract solutions diluted to a final concentration of 1 mM H_2_O_2_ and incubated for 24 h. Cell viability was then assessed using the CCK-8 assay. Additionally, intracellular levels of superoxide dismutase (SOD) and glutathione (GSH) were quantified in L929s following H_2_O_2_-induced oxidative stress (SOD Assay Kit, S0101; GSH Assay Kit, S0053; Beyotime).

ROS Detection: Intracellular reactive oxygen species (ROS) levels were detected using a green ROS fluorescent probe 2′,7′-dichlorodihydrofluorescein diacetate (DCFH-DA, S0033, Beyotime). L929s (3 × 10^4^ cells/well) were seeded in 48-well plates overnight. After treatment with 1 mM H_2_O_2_ and cryogel extract solutions for 24 h, the cells were washed three times with PBS and stained with 10 μM DCFH-DA for 30 min. Cellular ROS levels were then visualized using CLSM. Additionally, flow cytometry was employed to quantitatively assess intracellular ROS levels. Cells were seeded and treated in 6-well plates, stained with DCFH-DA, and subsequently analyzed by flow cytometry. Parallel experiments were performed to ensure reproducibility and statistical reliability.

#### 4.4.12 *In vitro* anti-inflammatory effect

DMEM extracts of each cryogel were prepared and used to treat Raw 264.7 cells. Cells (7 × 10^5^ cells/well) were seeded in 6-well plates and incubated overnight for attachment. After that, cells were treated with the extracts containing 1 μg/mL lipopolysaccharide (LPS, Thermo Fisher Scientific, L2880) for 24 h. DMEM with LPS served as the positive Control. After treatment, supernatants were collected for ELISA (Mouse TNF-α ELISA Kit, EM0183; Mouse IL-6 ELISA Kit, EM0121; Mouse IL-10 ELISA Kit, EM0100; FineTest) analysis of TNF-α, IL-6, and IL-10, and nitric oxide (NO) levels were measured using a kit (S0021, Beyotime). Cells were stained with anti-CD86 (105008, BioLegend) and anti-CD206 (141714, BioLegend) antibodies, followed by flow cytometric analysis.

### 4.5 Animal study

#### 4.5.1 *In vivo* full-thickness skin defect diabetes model

The animal experiments were approved by the Animal Ethics Committee of the Wenzhou Institute, University of Chinese Academy of Sciences (WIUCAS24072203). Female Sprague-Dawley (SD) rats, aged 8 weeks (weight: 200–250 g), were purchased from Beijing Vital River Laboratory Animal Technology Co., Ltd. (Beijing, China). Prior to modeling, the rats were fasted for 16 h with ad libitum access to water. A streptozotocin (STZ) working solution was prepared at a concentration of 10 mg/mL in a 0.1 M citric acid/sodium citrate buffer (pH 4.5). The rats were weighed and intraperitoneally injected with a single dose of 65 mg/kg of the STZ working solution. Four hours post-injection, a 20% glucose solution was administered by oral gavage. Blood glucose levels were measured on the third day after the injection, and rats with blood glucose levels exceeding 16.7 mmol/L were considered successfully diabetic and selected for further experiments. The rats were then randomly assigned into four groups: Control (PBS), BSAMA, HSAMA, and PlasmaMA.

After anesthetizing the rats with isoflurane, the hair on their backs was shaved. Full-thickness circular skin defects (Φ 6 × 2 mm^3^) were created on both sides of the back. The prepared cryogel samples were then placed onto the wound sites. Photographs were taken immediately after implantation (day 0) and at days 3, 7, and 14 to monitor wound healing. At these respective time points, wound tissues were harvested for histological analysis.

#### 4.5.2 *In vivo* implantation model

Cryogel samples (Φ 3 × 1 mm^3^) were prepared for implantation. Following isoflurane anesthesia, a longitudinal incision (approximately 10 mm in length) was made along the spine at the intersection of the scapula and vertebral column. Each cryogel sample was implanted into the resulting subcutaneous pocket, and the incision was closed using absorbable sutures (6-0). Skin tissue samples were harvested on post-implantation days 7, 14 and 21 for histological and biochemical analysis.

#### 4.5.3 Rat histological assessment

Following euthanasia, skin wound tissues were harvested and processed for histological analysis. Hematoxylin-eosin (H&E) and Masson's trichrome staining were performed to evaluate tissue morphology and collagen deposition, respectively. Additionally, immunofluorescence staining for CD31 (AF3628, R&D Systems/biotechne) was carried out to assess vascularization. Primary antibodies against CD68 (83014-5-RR), CD86 (30691-1-AP), CD206 (18704-1-AP), iNOS (18985-1-AP), and Arg-1 (16001-1-AP) (Proteintech, Wuhan, China) were applied for macrophage infiltration and polarization analysis. Immunohistochemical staining for TNF-α (60291-1-Ig), IL-10 (60269-1-Ig), Col-I (66761-1-Ig), Col-III (68320-1-Ig), and VEGF (66828-1-Ig) (Proteintech) was performed to assess inflammation, extracellular matrix deposition, and angiogenesis.

### 4.6 Statistical analysis

Experimental data were analyzed statistically and were presented as mean ± standard deviation (SD). One-way ANOVA and Student's t-test were used to assess differences between groups. A p-value of less than 0.05 was considered statistically significant.

## Ethical approval and informed consent statement

The human plasma samples used in this study were commercially obtained from Kejing Biological Technology Co., Ltd. According to the supplier's statement, the samples were collected with informed consent from the donors and were fully anonymized prior to distribution. No identifiable personal information was accessible to the researchers.

As this study did not involve direct human participation, fresh sampling, or access to personal data, ethical approval was exempted in accordance with institutional guidelines.

All experimental procedures were carried out in accordance with the principles of the Declaration of Helsinki.

## Author information

All authors have approved the final version of this manuscript.

## CRediT authorship contribution statement

**Yueming Zhao:** Investigation, Methodology, Project administration, Visualization, Writing – original draft, Writing – review & editing. **Jiajun Hu:** Investigation, Methodology, Project administration, Visualization, Writing – review & editing. **Kairui Duan:** Methodology, Writing – original draft. **Tingting Li:** Investigation, Validation. **Mian Lin:** Investigation, Validation. **Bae Hoon Lee:** Conceptualization, Funding acquisition, Investigation, Supervision, Writing – original draft, Writing – review & editing.

## Declaration of competing interest

The authors declare that they have no known competing financial interests or personal relationships that could have appeared to influence the work reported in this paper.

## Data Availability

Data will be made available on request.
