# Supplementary material for: Engineered human plasma-derived cryogels as a multifunctional scaffold to promote diabetic wound healing
Source: Mater Today Bio. 2026 Apr 22;38:103151. doi: 10.1016/j.mtbio.2026.103151 (PMC13141794; doi:10.1016/j.mtbio.2026.103151)
Supplement: Multimedia component 1 [file mmc1.docx]

Electronic Supplementary Information

for

**Engineered Human Plasma-Derived Cryogels as a Multifunctional Scaffold to Promote Diabetic Wound Healing**

Yueming Zhao^a, b, 1^, Jiajun Hu^c, 1^, Kairui Duan^b^, Tingting Li^b^, Mian Lin^b^, Bae Hoon Lee^b, *^

^a^ School of Pharmaceutical Sciences, Zhejiang Chinese Medical University, Hangzhou, Zhejiang 311402, China

^b^ Wenzhou Institute, University of Chinese Academy of Sciences, Wenzhou, Zhejiang 325011, China

^c^ Department of Periodontics, School & Hospital of Stomatology, Wenzhou Medical University, Wenzhou, Zhejiang 325027, China

**Table S1.** Cryogelation of PlasmaMA at different concentrations.

| **Compositions** | **2% PlasmaMA** | **3% PlasmaMA** | **4% PlasmaMA** |
| --- | --- | --- | --- |
| **PlasmaMA (mg)** | 20 | 30 | 40 |
| **PBS (μL)** | 945 | | |
| **10% APS (μL)** | 50 | | |
| **20% TEMED (μL)** | 5 | | |
| **Dry** | 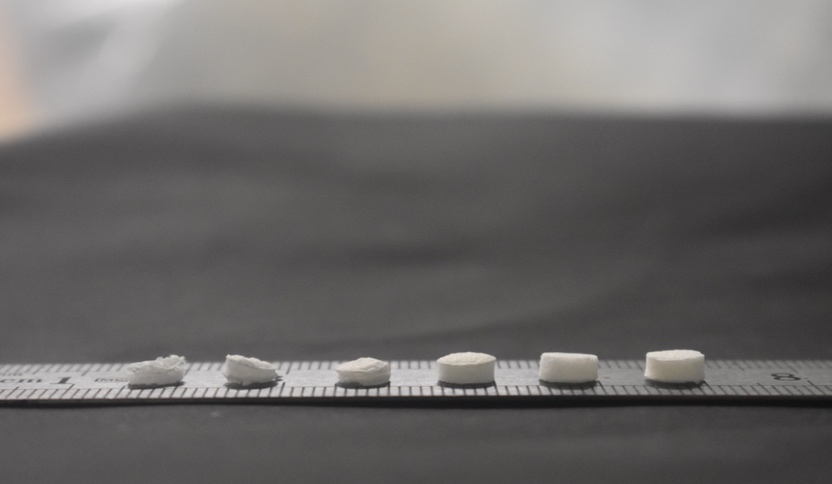  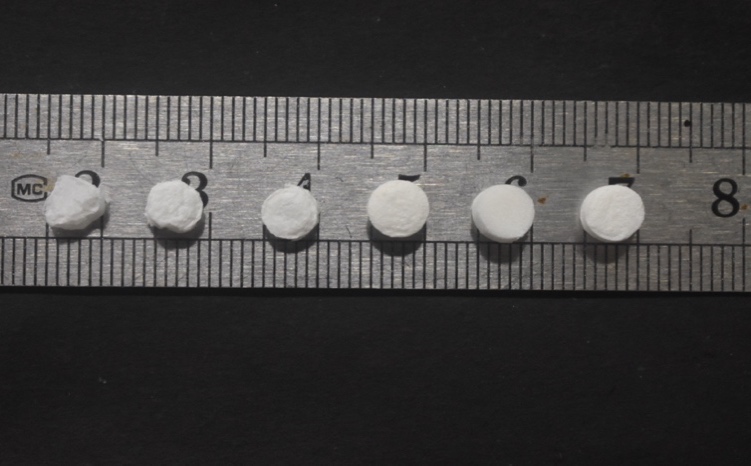 | | |
| **Hydrated** | **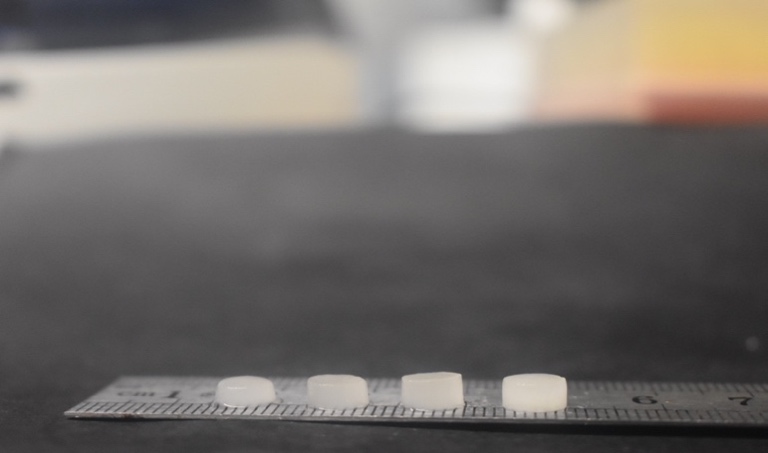**  **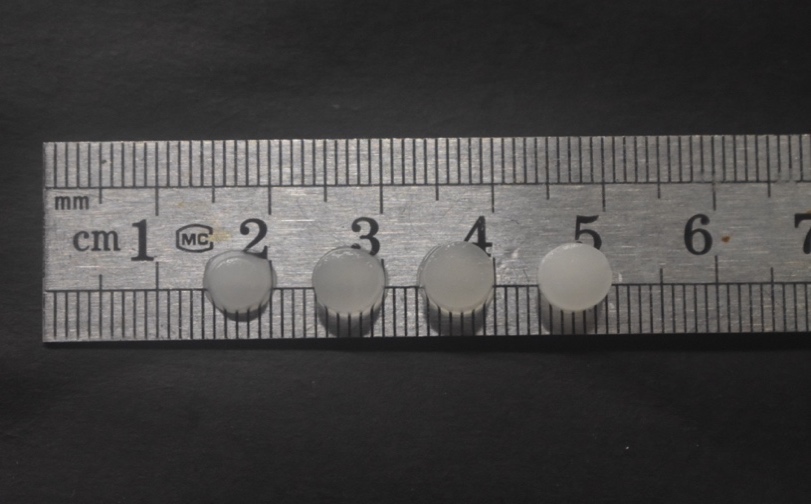** | | |

**Table S2.** The compositions of BSAMA, HSAMA, and PlasmaMA cryogels (3%).

| **Compositions** | **BSAMA** | **HSAMA** | **PlasmaMA** |
| --- | --- | --- | --- |
| **BSAMA/HSAMA/PlasmaMA (mg)** | 30 | 30 | 30 |
| **PBS (µL)** | 945 | 945 | 945 |
| **10% APS (µL)** | 50 | 50 | 50 |
| **20% TEMED (µL)** | 5 | 5 | 5 |

**Table S3.** Sequences of primers used in qRT-PCR analysis.

| **Gene** | **Forward primer (5'-3')** | **Reverse primer (5'-3')** |
| --- | --- | --- |
| **GAPDH (human)** | GTGGACCTGACCTGCCGTCTAG | GAGTGGGTGTCGCTGTTGAAGTC |
| **β-Actin (mouse)**  **VEGF** | GTGCTATGTTGCTCTAGACTTCG  TTGCCTTGCTGCTCTACCT | ATGCCACAGGATTCCATACC  CATCCATGAACTTCACCACTTC |
| **EGF** | CAGGTGGTGAAGTTGATCTA | ACAGGAGCACAGTCATCT |
| **TGF-β1** | CAAGTTCAAGCAGAGTACAC | TGAGGTATCGCCAGGAAT |
| **GDF-15**  **Col-I** | CTGGTGTTGCTGGTGCTCTCG  TTCGGAGGAGAGTCAGGAAG | TCGGAATCTGGAGTCTTCGGAGTG  CAGCAACACAGTTACACAAGG |
| **HGF** | TGTGCTGGGGCTGAAAAGATTGG | CCACGACCAGGAACAATGACACC |
| **TGF-α** | TGTTCGCTCTGGGTATTGTGTTGG | GGAAGCAGAACTGAGTGTGGGAAT |
| **BMPR2** | CACCTCCTGACACAACACCACTC | ATGCTGCTGCCTCCATCATGTTC |
| **Col-Ⅱα1** | GGTCCTCCTGGTCCTGGCATC | CGTGCTGTCTCAAGGTACTGTCTG |


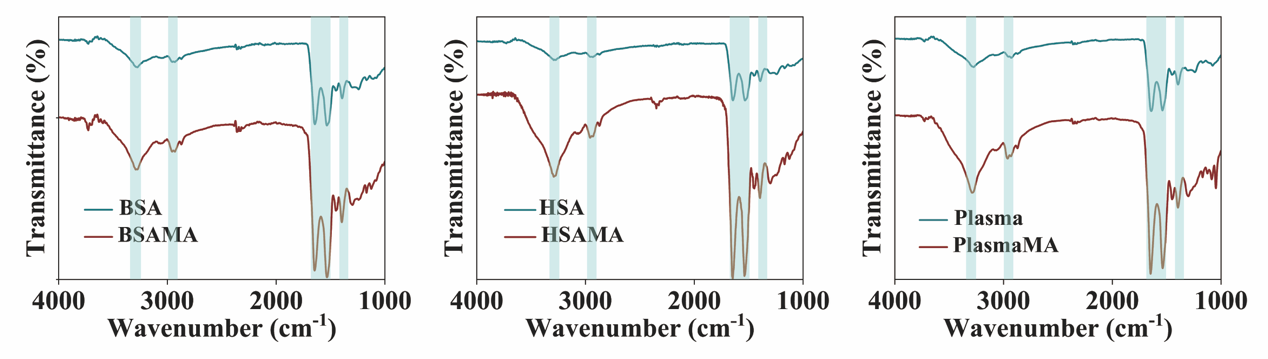


**Fig. S1.** FT-IR Spectra of BSA, BSAMA, HSA, HSAMA, Plasma, and PlasmaMA.


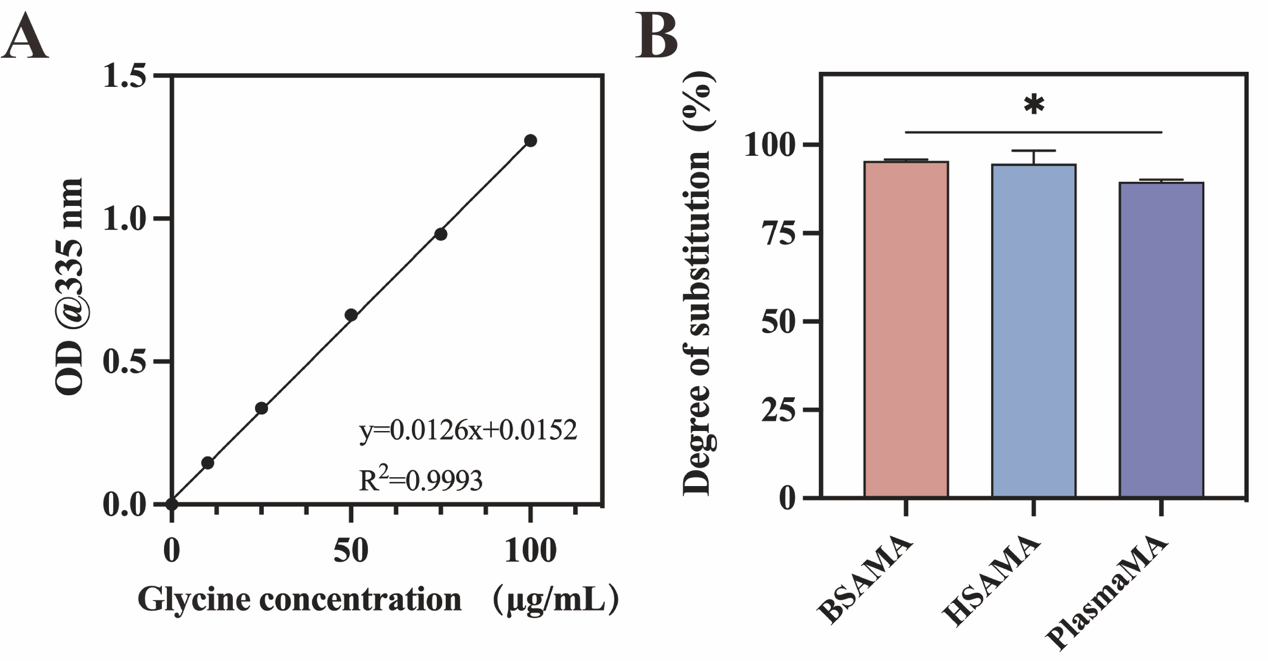


**Fig. S2.** (A) Glycine standard curve in TNBS assay. (B) Degree of substitution of BSAMA, HSAMA, and PlasmaMA determined by the TNBS assay. * *p* < 0.05, (n = 3, mean ± SD).


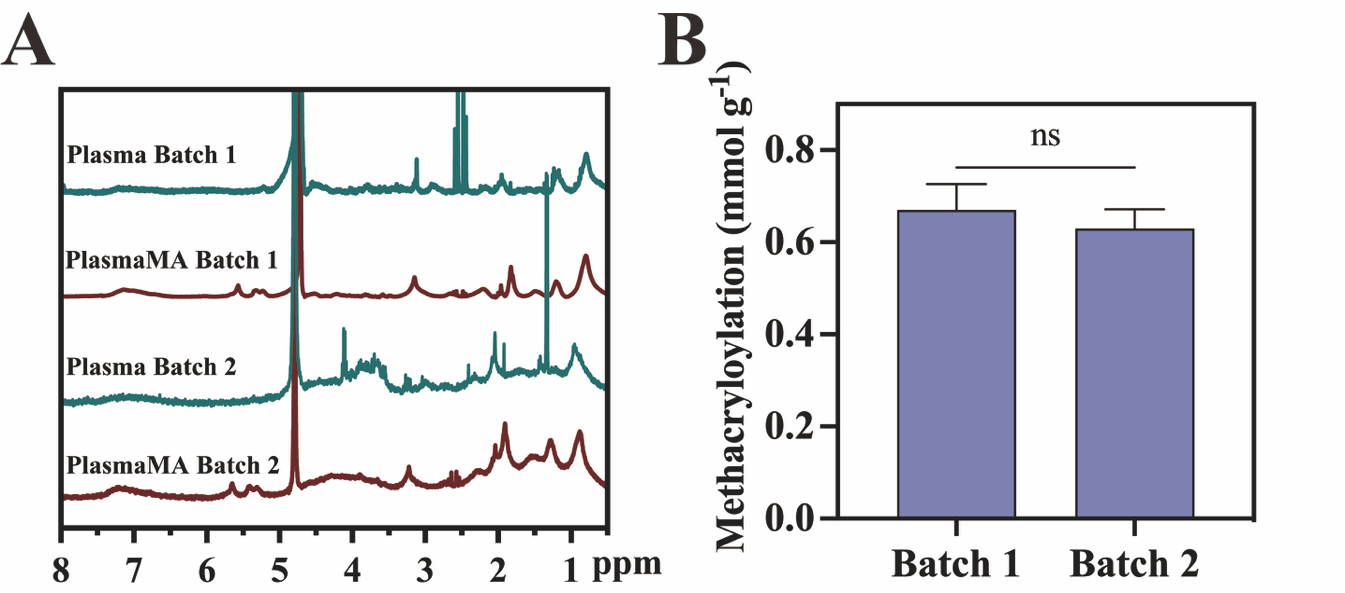


**Fig. S3.** NMR characterization of different batches of PlasmaMA. (A) ^1^H-NMR spectra of two batches of Plasma and their corresponding PlasmaMA. (B) Methacryloylation degree of each batch calculated from peak integration. ns denotes ‘not significant’ (n = 3, mean ± SD).


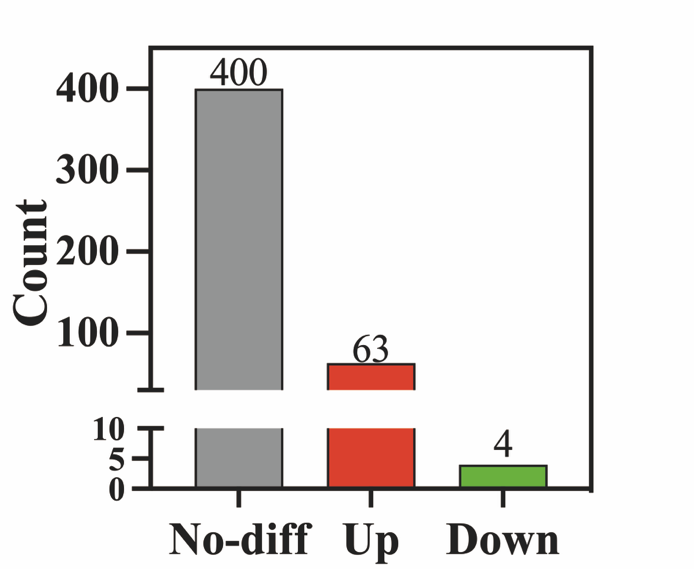


**Fig. S4.** Quantitative summary of upregulated and downregulated proteins after methacryloylation based on the volcano plot.


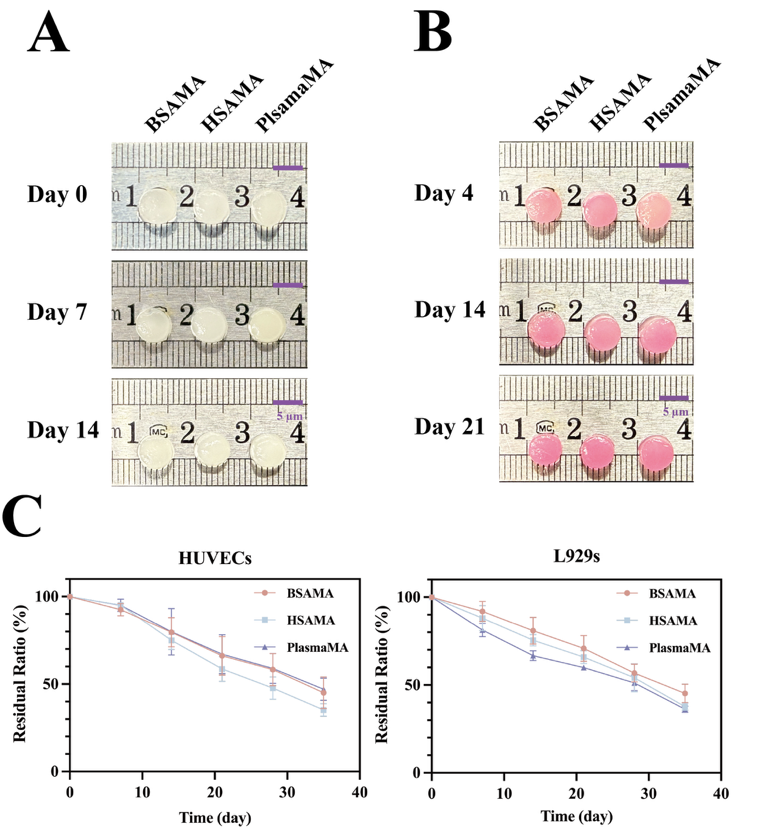


**Fig. S5.** (A) Images of BSAMA, HSAMA, and PlasmaMA cryogels in PBS on days 0, 7, and 14, incubated and agitated at 37 °C and 150 rpm. (B) Images of cryogels co-cultured with L929s on days 4, 14, and 21. (C) Residual mass ratios of BSAMA, HSAMA, and PlasmaMA cryogels co-cultured with HUVECs and L929s for 35 days, respectively (n = 3, mean ± SD).


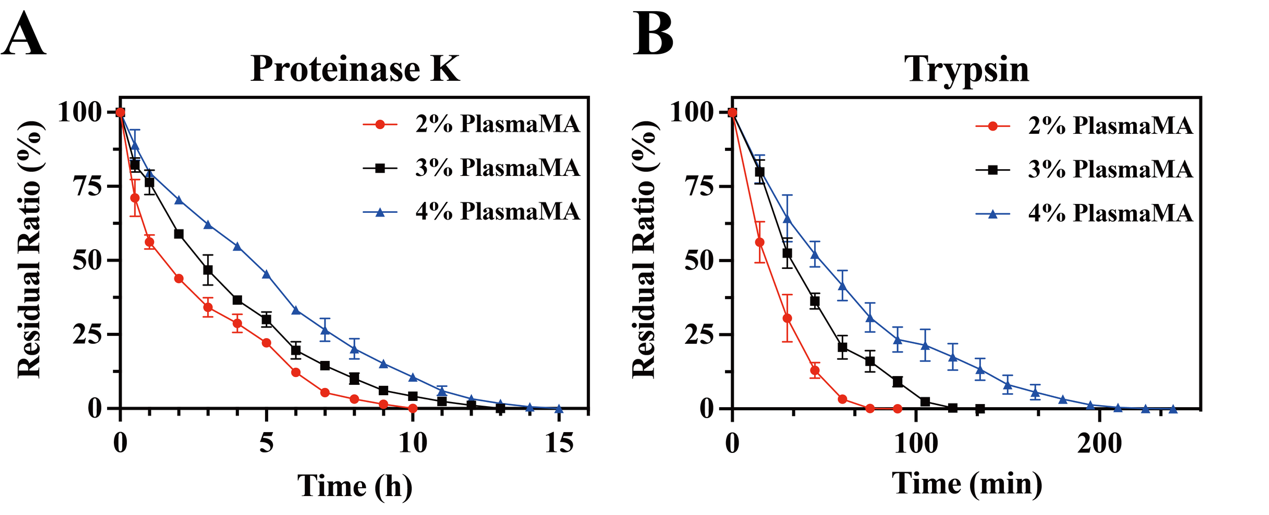


**Fig. S6.** Residual mass ratios of 2%, 3%, and 4% PlasmaMA cryogels incubated in 0.01 mg/mL Proteinase K (A) and 0.1% Trypsin (B) (n = 3, mean ± SD).


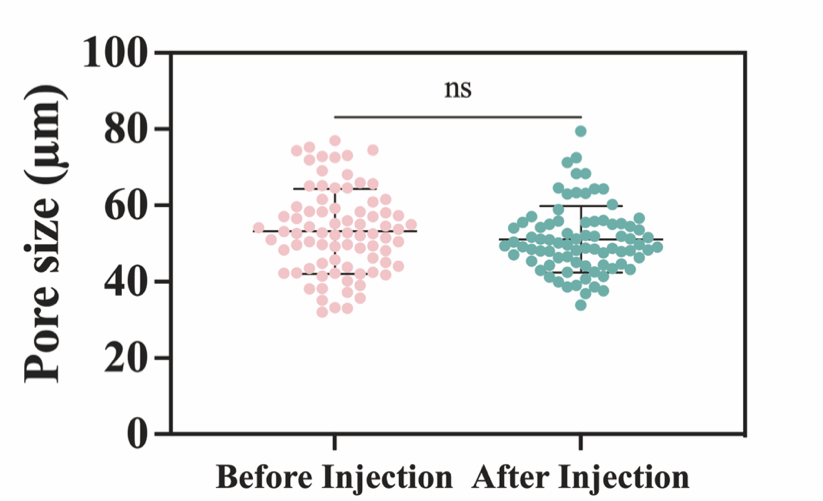


**Fig. S7.** Quantitative analysis of the pore size of PlasmaMA cryogel before and after injection. ns denotes ‘not significant’ (n = 75, mean ± SD).


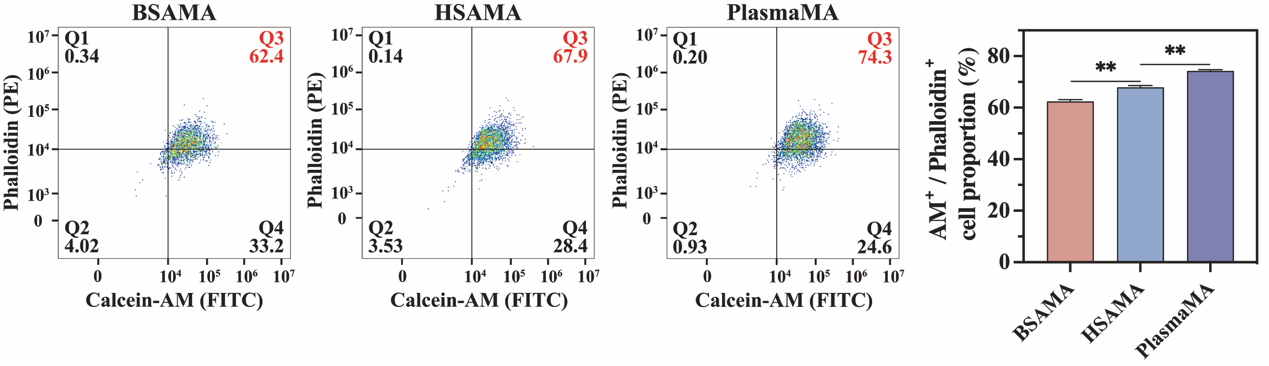


**Fig. S8.** Flow cytometry of Calcein-AM and Rhodamine-Phalloidin-stained L929s on cryogels after 4 h (n = 3). * *p* < 0.05, and ** *p* < 0.01 (n = 3, mean ± SD); ns denotes ‘not significant’.


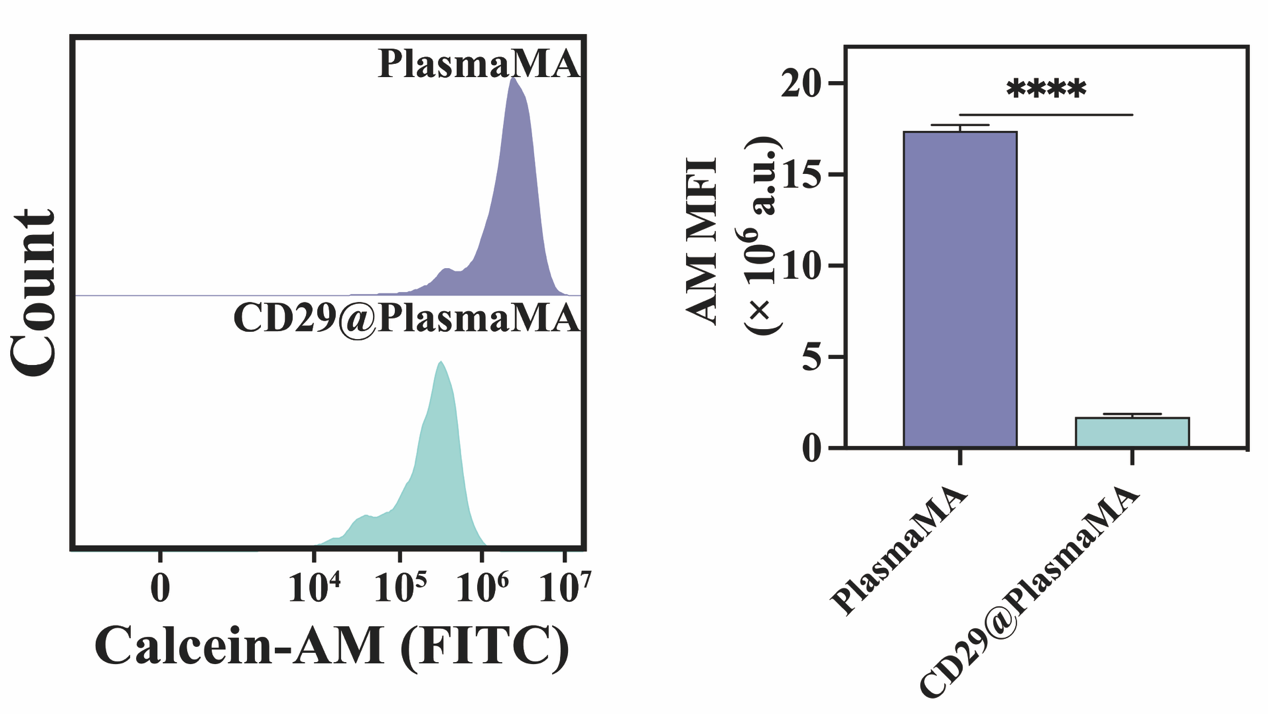


**Fig. S9.** Effect of CD29 blocking on HUVECs adhesion to 3D PlasmaMA cryogels. Viable adherent cells were stained with Calcein-AM and quantitatively analyzed by flow cytometry to assess the impact of integrin β1 (CD29) blockade on cell adhesion. * *p* < 0.05, ** *p* < 0.01, *** *p* < 0.001, and **** *p* < 0.0001 (n = 3, mean ± SD); ns denotes ‘not significant’.


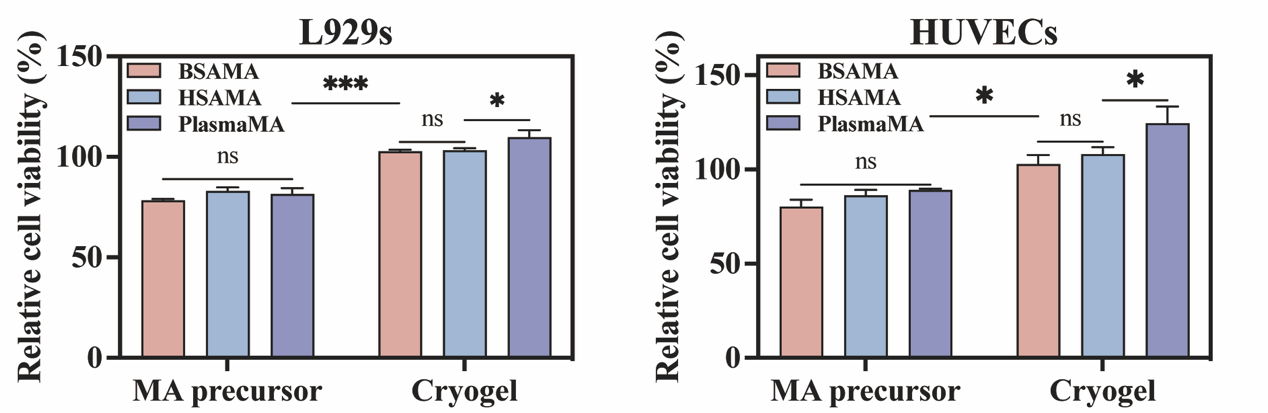


**Fig. S10.** Improved cytocompatibility after cryogel formation. Cell viability of L929s and HUVECs after 24 h incubation with extracts from MA precursors and the corresponding cryogels, as evaluated by the CCK-8 assay. Cell viability was normalized to untreated control (set as 100%). * *p* < 0.05, ** *p* < 0.01, and *** *p* < 0.001 (n = 3, mean ± SD); ns denotes ‘not significant’.


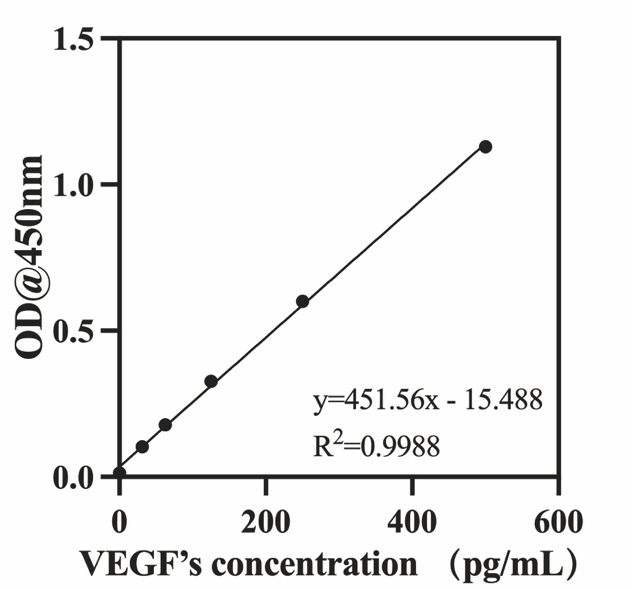


**Fig. S11.** Calibration curve of VEGF (linear regression correlation coefficient R^2^ = 0.9988).


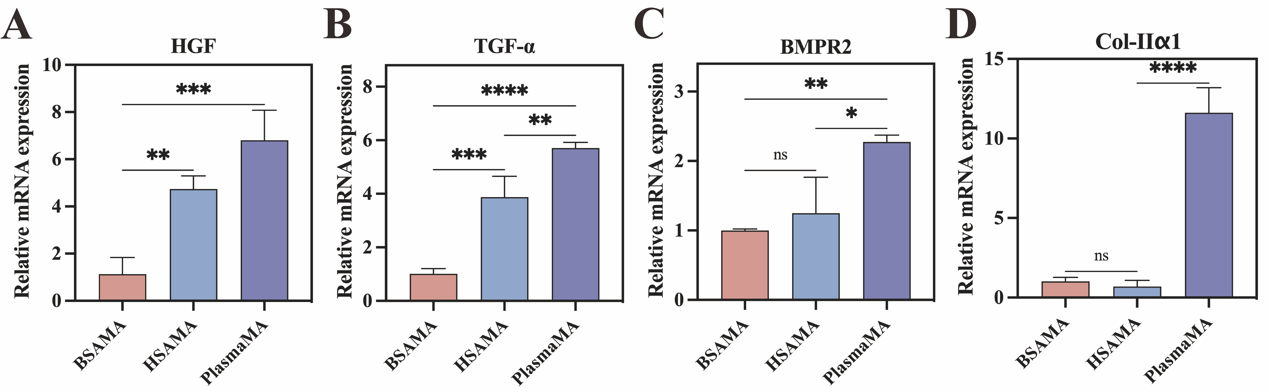


**Fig. S12.** HUVECs and L929s were separately co-cultured with cryogels for 4 days, and the gene expressions of (A) HGF, (B) TGF-α, (C) BMPR2, and (D) Col-IIα1 were measured. * *p* < 0.05, ** *p* < 0.01, and *** *p* < 0.001 (n = 3, mean ± SD ); ns denotes ‘not significant’.


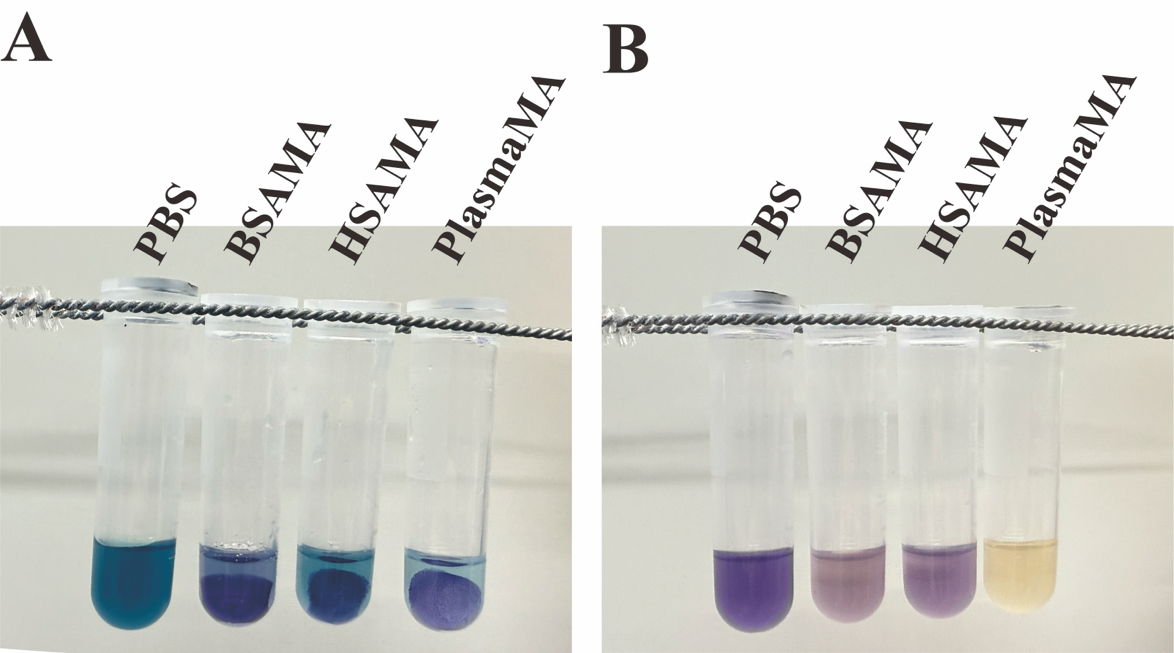


**Fig. S13.** Representative images showing colorimetric differences among groups in the ABTS (A) and DPPH (B) radical scavenging assays.


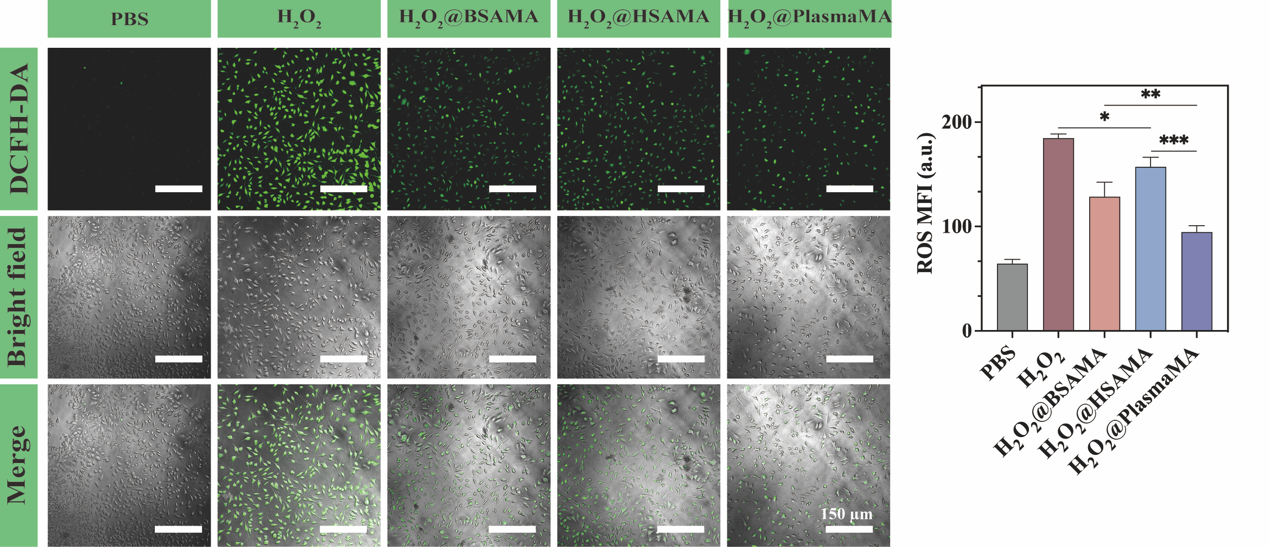


**Fig. S14.** CLSM images and corresponding fluorescence intensity analysis of intracellular ROS levels in L929s. Scale bar: 150 µm. * *p* < 0.05, ** *p* < 0.01, and *** *p* < 0.001 (n = 3, mean ± SD); ns denotes ‘not significant’.


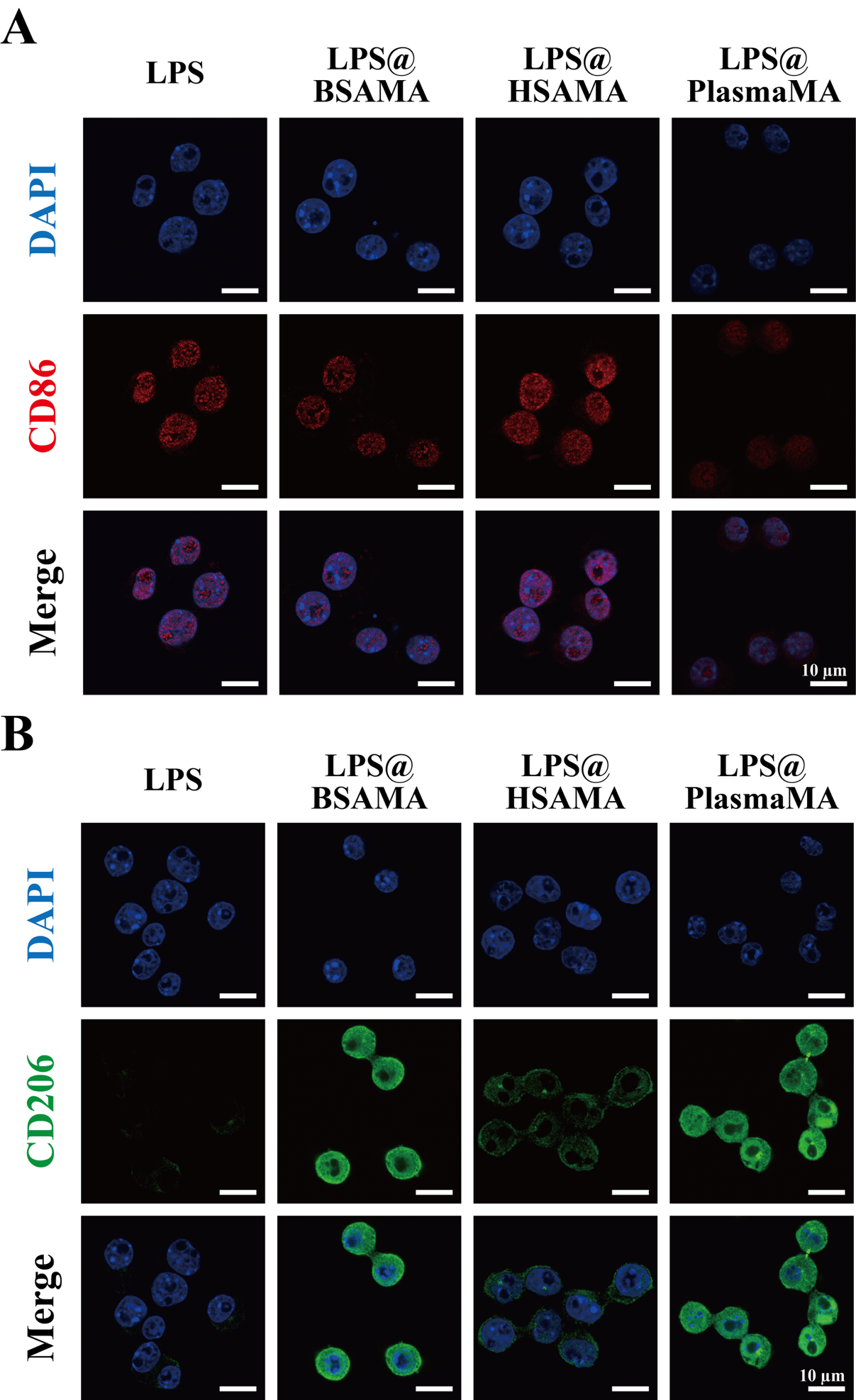


**Fig. S15.** Immunofluorescence staining of (A) CD86 and (B) CD206 in RAW 264.7 cells. Nuclei were stained with DAPI (blue), CD86 is shown in red, and CD206 is shown in green. Scale bar: 10 µm.
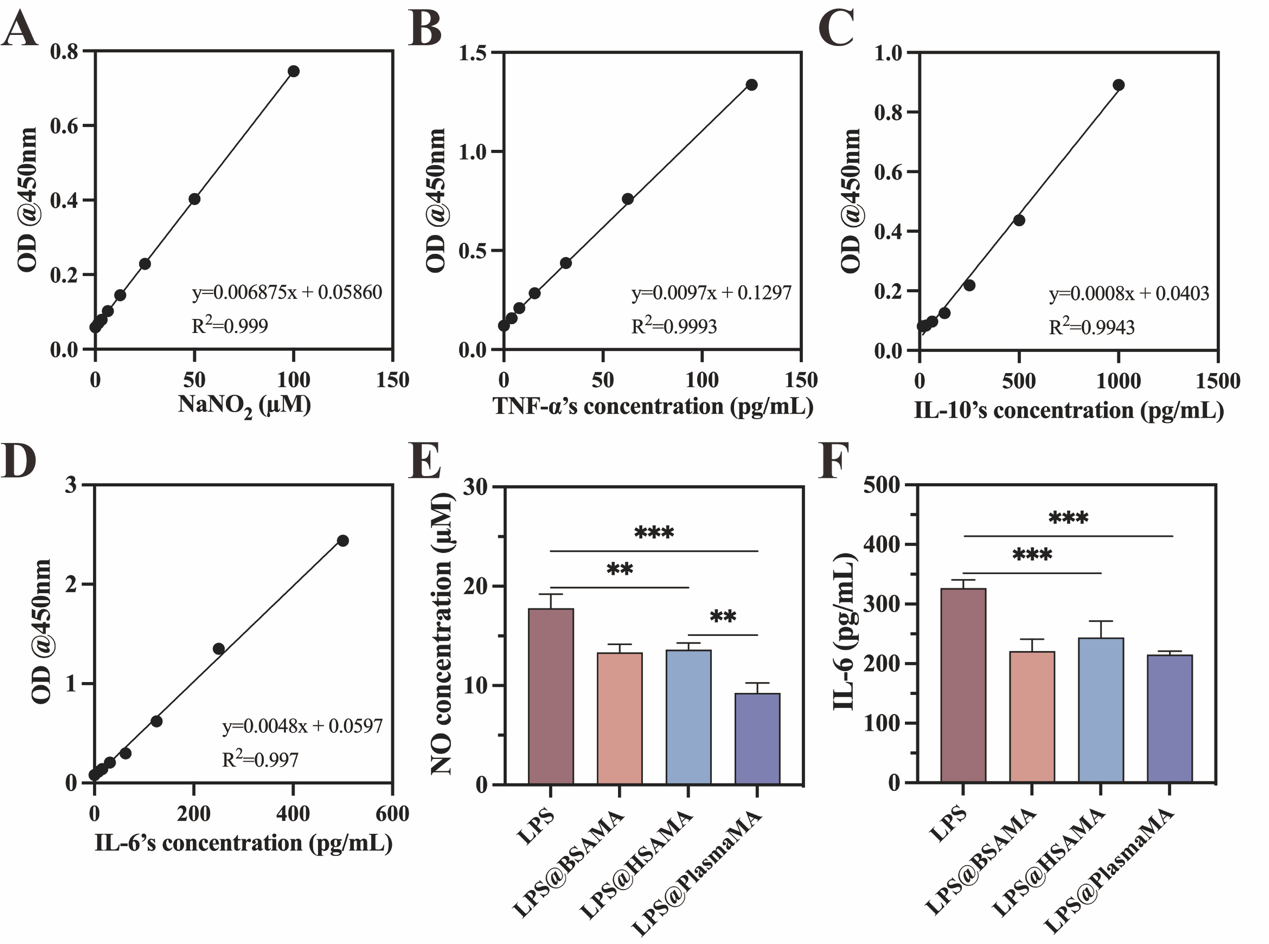


**Fig. S16.** Calibration curves of (A) NaNO₂ (R^2^ = 0.999), (B) TNF-α (R^2^ = 0.9993), (C) IL-10 (R^2^ = 0.9943), and (D) IL-6 (R^2^ = 0.997) based on linear regression. (E) Nitric oxide (NO) production was quantified by Griess assay and (F) IL-6 levels measured by ELISA in LPS-stimulated Raw 264.7 cells. * *p* < 0.05, ** *p* < 0.01, and *** *p* < 0.001 (n = 3, mean ± SD); ns denotes ‘not significant’.


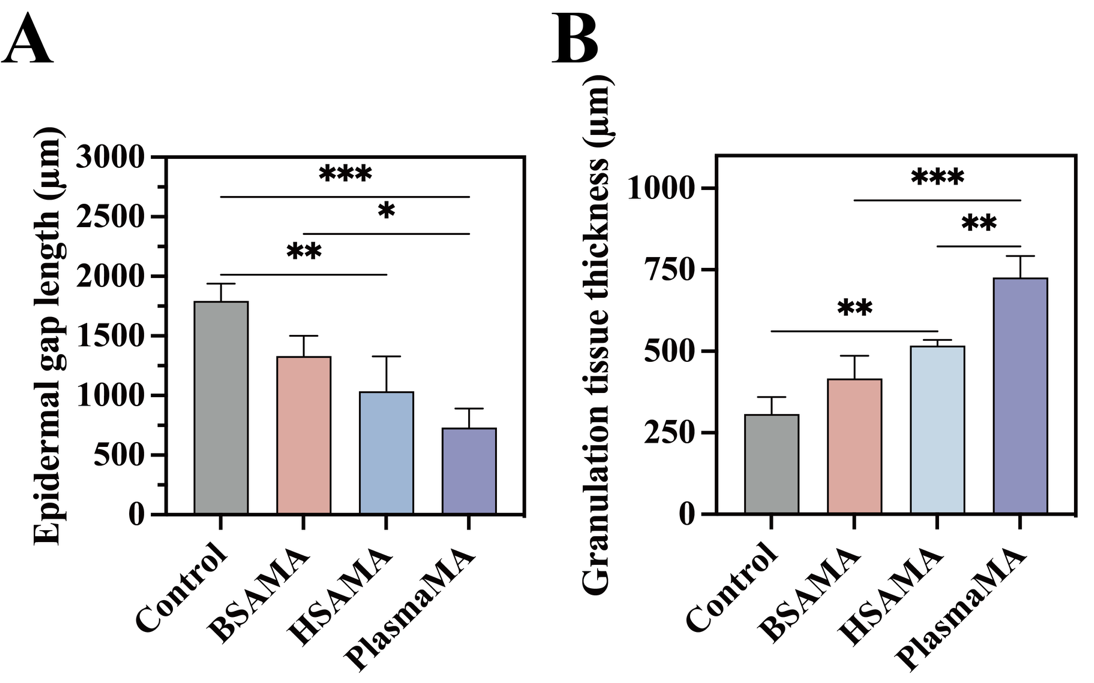


**Fig. S17.** The epidermal gap length (A) and granulation tissue thickness (B) were quantitatively analyzed and compared at day 14. * *p* < 0.05, ** *p* < 0.01, and *** *p* < 0.001 (n = 3, mean ± SD); ns denotes ‘not significant’.


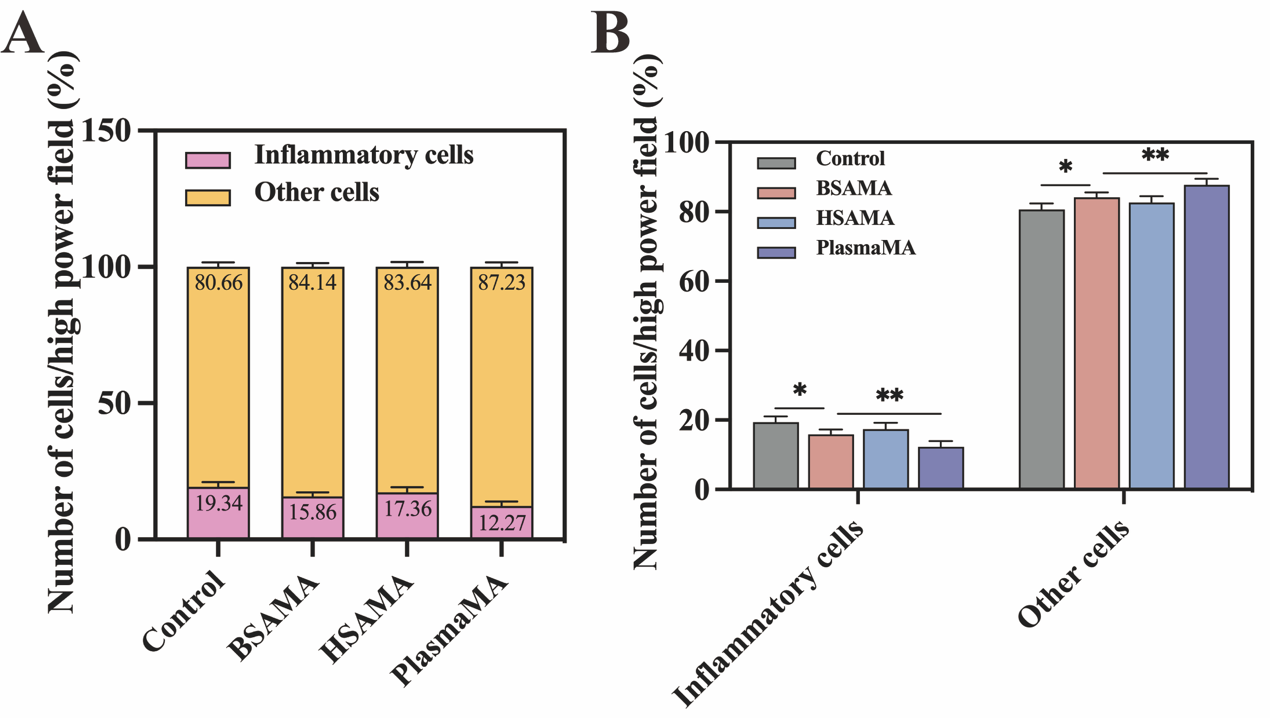


**Fig. S18.** The proportions of inflammatory cells and other cells were quantitatively analyzed and compared at day 3. * *p* < 0.05, and ** *p* < 0.01 (n = 5, mean ± SD); ns denotes ‘not significant’.


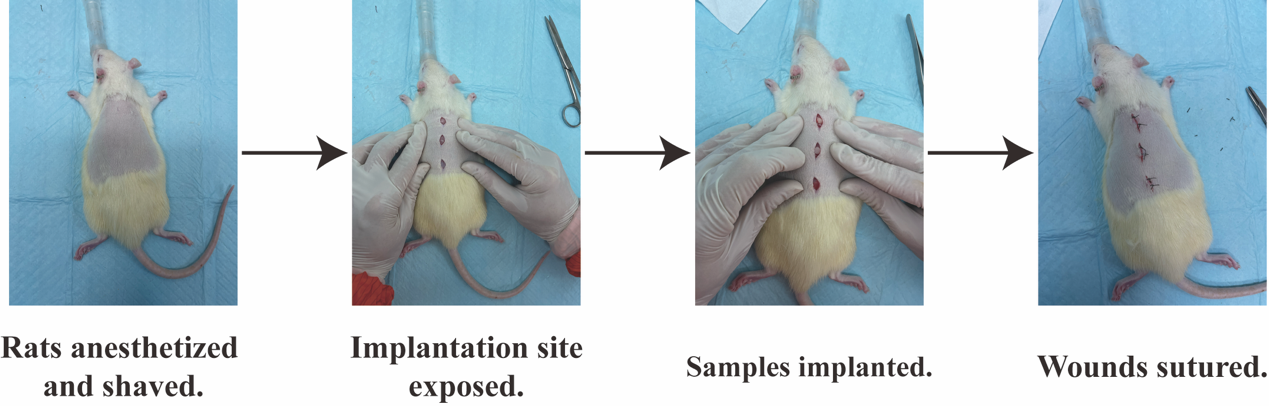


**Fig. S19.** Subcutaneous implantation procedure.


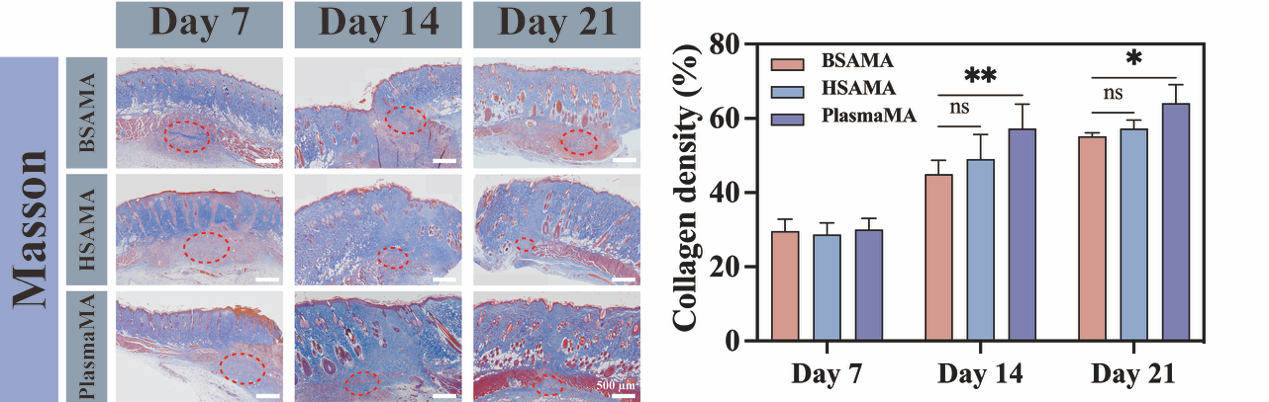


**Fig. S20.** Masson’s trichrome staining of tissue sections harvested on days 7, 14, and 21 following subcutaneous implantations of BSAMA, HSAMA, and PlasmaMA cryogels (Scale bar: 500 μm). The red circle marks the approximate contour of the residual cryogel. * *p* < 0.05, ** *p* < 0.01 (n = 4, mean ± SD); ns denotes ‘not significant’.


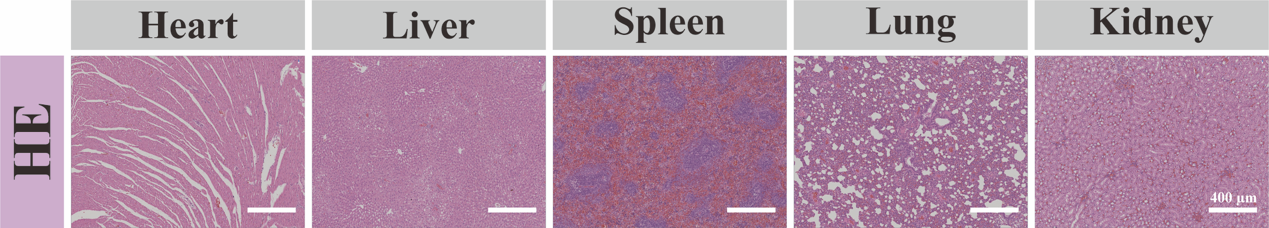


**Fig. S21.** H&E images of main organs after subcutaneous implantation with samples at 14 days (Scale bar: 400 μm).


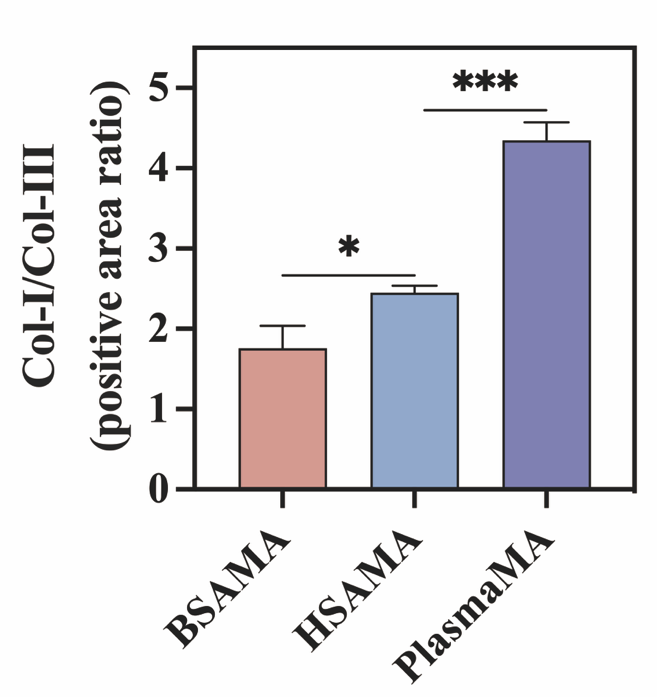


**Fig. S22.** Col-I/Col-III ratio on day 21 in the subcutaneous implantation model, as assessed by IHC staining. * *p* < 0.05, ** *p* < 0.01, and *** *p* < 0.001 (n = 3, mean ± SD); ns denotes ‘not significant’.


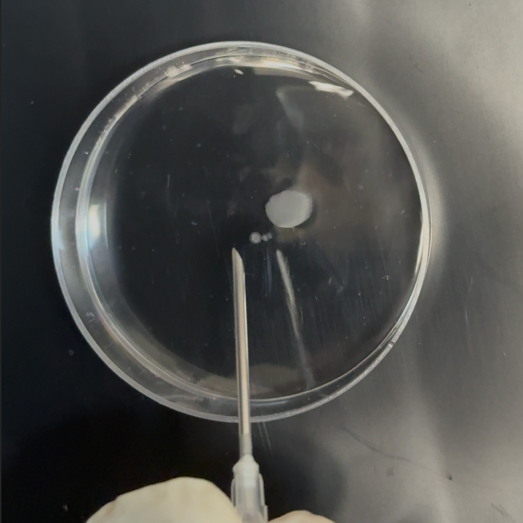


**Video S1.** Within 5 seconds of injection via a 16-gauge needle, the PlasmaMA cryogel demonstrates rapid recovery of its original morphology.
